# USP9X deubiquitylating enzyme maintains RAPTOR protein levels, mTORC1 signalling and proliferation in neural progenitors

**DOI:** 10.1038/s41598-017-00149-0

**Published:** 2017-03-24

**Authors:** Caitlin R. Bridges, Men-Chee Tan, Susitha Premarathne, Devathri Nanayakkara, Bernadette Bellette, Dusan Zencak, Deepti Domingo, Jozef Gecz, Mariyam Murtaza, Lachlan A. Jolly, Stephen A. Wood

**Affiliations:** 1 Eskitis Institute for Drug Discovery, Griffith University, Brisbane, Queensland Australia; 2 School of Molecular and Biomedical Science, University of Adelaide, Adelaide, South Australia Australia; 3 Robinson Institute, School of Paediatrics and Reproductive Health, University of Adelaide, Adelaide, South Australia Australia

## Abstract

USP9X, is highly expressed in neural progenitors and, essential for neural development in mice. In humans, mutations in *USP9X* are associated with neurodevelopmental disorders. To understand *USP9X*’*s* role in neural progenitors, we studied the effects of altering its expression in both the human neural progenitor cell line, ReNcell VM, as well as neural stem and progenitor cells derived from Nestin-*cre* conditionally deleted *Usp9x* mice. Decreasing USP9X resulted in ReNcell VM cells arresting in G0 cell cycle phase, with a concomitant decrease in mTORC1 signalling, a major regulator of G0/G1 cell cycle progression. Decreased mTORC1 signalling was also observed in *Usp9x*-null neurospheres and embryonic mouse brains. Further analyses revealed, (i) the canonical mTORC1 protein, RAPTOR, physically associates with Usp9x in embryonic brains, (ii) RAPTOR protein level is directly proportional to USP9X, in both loss- and gain-of-function experiments in cultured cells and, (iii) USP9X deubiquitlyating activity opposes the proteasomal degradation of RAPTOR. EdU incorporation assays confirmed Usp9x maintains the proliferation of neural progenitors similar to Raptor-null and rapamycin-treated neurospheres. Interestingly, loss of Usp9x increased the number of sphere-forming cells consistent with enhanced neural stem cell self-renewal. To our knowledge, USP9X is the first deubiquitylating enzyme shown to stabilize RAPTOR.

## Introduction

The proliferation, self-renewal and differentiation of neural stem cells (NSCs), and their closely related derivatives, neural progenitors (NPs), in both the developing and adult nervous systems, are tightly controlled by both extrinsic and intrinsic signals. Extrinsic signals, derived from the stem cell niche or embryonic organising centres, need to be recognised, relayed internally, coordinated and interpreted to ultimately produce a precise cellular response. Cell polarity, adhesion, cycle length and migration all influence the response to extrinsic signals. Concerning the cell cycle, the G0 and G1 phases are particularly involved in dictating the fate of a NP^1^. Specifically, it is the length of the G1 phase that regulates progenitor differentiation. Cells that linger in these phases have increased longevity and retain the capacity to self-renew^1^. Multiple extrinsic signals influence NP cell cycle progression and the intracellular mammalian target of rapamycin (mTOR) pathway responds to many of these growth factors by coordinating an appropriate RNA and protein synthesis response and, as such, is a major regulator of G0/G1 cell cycle progression. mTOR is an ubiquitous protein kinase found in two complexes, mTORC1 and mTORC2. The mTORC1 complex, which consists of mTOR, regulatory-associated protein of mTOR (RAPTOR) and mammalian lethal with SEC13 protein 8 (MLST8), responds to growth factor stimulation and links this, along with nutrient availability, to cell growth and division^2, 3^. In particular, mTORC1 response to growth factor stimulation is required for cell cycle progression through the G1 phase of the cell cycle^2^.

mTOR is a critical regulator of NSC/NP function *in vivo* and *in vitro* balancing self-renewal, proliferation, differentiation and maturation^4^. Dysregulation of mTOR signalling gives rise to neurodevelopmental disorders^5, 6^. Hyperactivation of the mTOR pathway, due to loss of function mutations in the *TSC1* and *TSC2* genes, upstream inhibitors of mTOR, gives rise to Tuberous Sclerosis Complex (TSC). TSC is characterized by benign malformations comprised of aberrantly proliferating non-malignant cells of the tissue of origin. In the brain, these lesions are ectopic neurogenic compartments with enhanced proliferation of NPs and their subsequent premature differentiation^7^. Conversely loss of mTORC1 function results in decreased NP proliferation. Deletion of RAPTOR, an essential protein of the mTORC1 complex, from NPs of the dorsal telencephalon leads to decreased proliferation but not loss of self-renewal capacity^8^. Similarly, these cardinal features are seen in *ex*-*vivo* cultures of murine NSC/NPs grown as free-floating aggregates, called neurospheres, lacking mTORC1 function: inhibition of mTORC1 signalling in neurospheres, by addition of rapamycin, also decreased proliferation of NPs without affecting the self-renewing capacity of the NSCs^9^. Therefore, mTOR signalling needs to be tightly balanced to maintain homeostasis in NPs.

Another protein well placed to integrate extrinsic signals with the intrinsic responses of NPs, is the ubiquitin-specific protease 9 located on the X-chromosome (USP9X). USP9X is a deubiquitylating enzyme highly expressed in adult and embryonic NPs *in vivo*, and *in vitro* ^10^. Altering *Usp9x* expression levels affects NP function. Moderately increased *Usp9x* expression in mouse embryonic stem cell-derived NPs *in vitro* promotes their self-renewal leading to a large increase in the number of NPs^11^. Conversely, Nestin-*cre* mediated deletion of *Usp9x* from all NPs of the mouse central nervous system disrupted their organisation in the ventricular and sub-ventricular zones and results in peri-natal lethality^12^. Deletion of *Usp9x* from the dorsal telencephalon only, is compatible with post-natal survival, but results in a dramatic 75% reduction in adult hippocampal size, suggesting NP proliferation is reduced^12^. Mutations in human *USP9X* are associated with several neurodevelopmental disorders including X-linked intellectual disability and autism^13^. In addition, mutations in Doublecortin that specifically disrupt its ability to interaction with USP9X, result in lissencephaly and severe epilepsy, further highlighting the importance of USP9X function for normal brain development^14^.

Recently, Usp9x has been implicated in mTOR signalling in C2C12 mouse muscle myoblasts^15^. Knockdown of *Usp9x* in these cells increased mTORC1 activity^15^. Epitope pull-down assays showed that Usp9x associated with mTOR, as well as RAPTOR and RICTOR, signature proteins of the mTORC1 and mTORC2 signalling complexes, respectively^15^. However altered expression of USP9X did not affect the level of mTOR protein in HEK293 cells. Here, we show that USP9X is a potent regulator of the mTORC1 signalling in NP/NSCs. Decreasing USP9X levels resulted in a rapid arrest of cultured NPs in G0/G1 of the cell cycle. Further we show that USP9X binds RAPTOR in the developing brain and maintains RAPTOR levels in cultured NPs suggesting RAPTOR is a critical USP9X substrate.

## Results

### USP9X depletion results in reduced neural progenitor number

**Figure 1:**
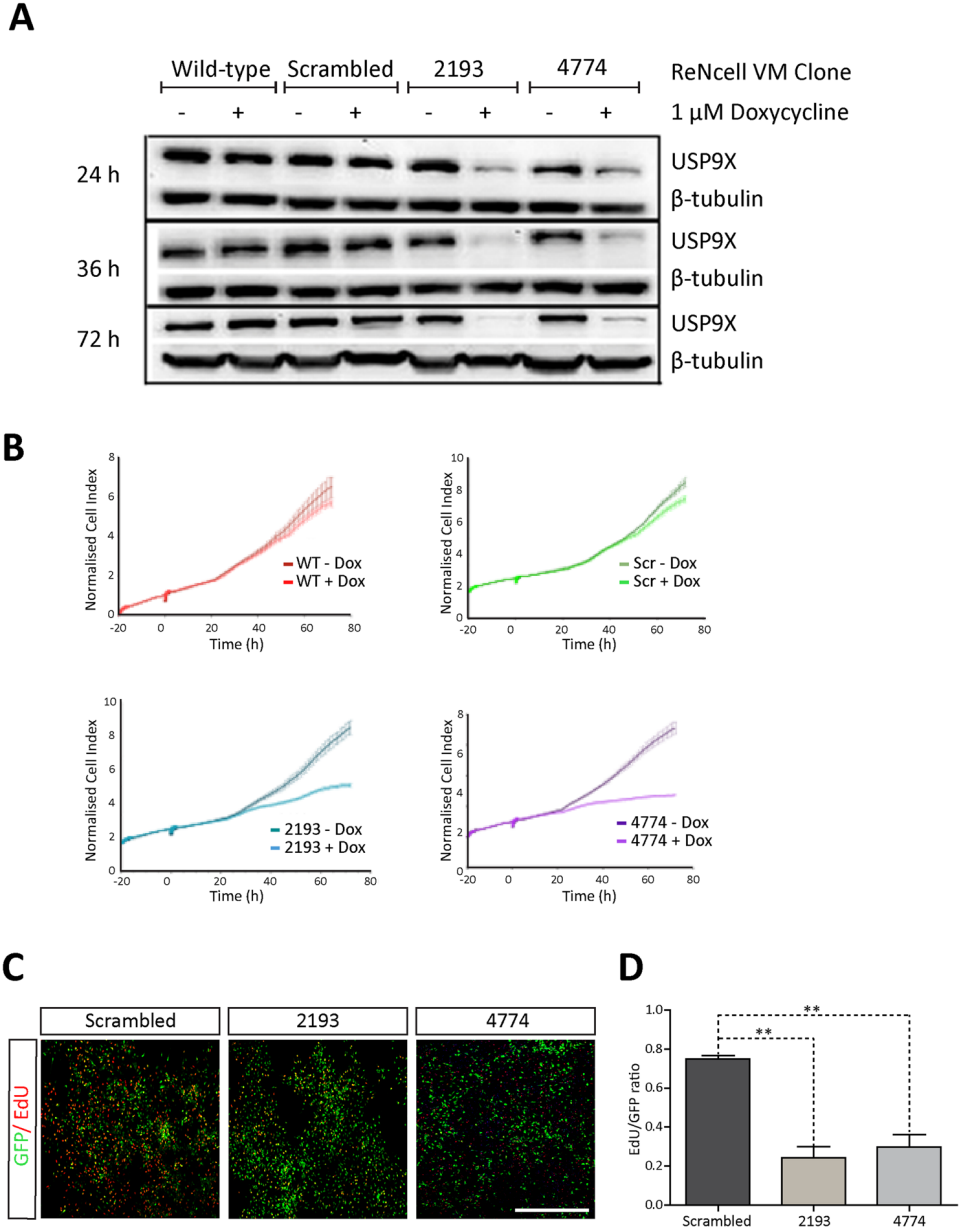
USP9X depletion reduces ReNcells VM proliferation. (**A**) Doxycycline treatment depleted USP9X protein levels after 24, 36 and 72 hours in ReNcell VM cells harbouring *USP9X*-targeted shRNA (2913 and 4774) but not a nonsense shRNA (Scrambled) or non-transduced cells (Wildtype). Blot representative of six biological replicates. Gels were run under the same experimental conditions. USP9X and β-tubulin proteins at expected molecular weights. (**B**) Reduced Cell index of ReNcell VM cells following addition of 1 μM Doxycycline (0 hour). xCELLigence real-time measurement of increasing electrical impedance plotted on “Y axis” (“Normalised Cell Index”). Cell Index of doxycycline treated (+) ‘2193’ ReNCell VM cells differed significantly from non-treated after 34 hours (p < 0.05), 35 hours (p < 0.01) and 36 hours (p < 0.001). After 72 hours, the cell index was approximately 50% of untreated 2193 cells. For ‘4774’ cells, the cell index differed significantly at 28 hours (p < 0.05), 29 hours (p < 0.01) and 30 hours (p < 0.001) after treatment. After 72 hrs, the reduction in cell index was approximately 61% that of untreated 4774 cells. Analysis was obtained by plotting t-values of the two way ANOVA analysis (versus time). Error bars represent standard deviation. wt: wildtype, scr: scrambled, +: 1 μM doxycycline treated, −: No doxycycline. Representative of two biological replicates. (**C**) ReNcell VM cells exposed to doxycycline for 72 hours were labelled with a 6 hour pulse of EdU, to mark cells undergoing DNA-synthesis in S-phase, before immunofluorescence staining for GFP (transduced cells) and EdU (Red). (**D**) Quantitation of cell counts revealed significantly fewer EdU-positive cells in doxycycline-treated 2193 and 4774 cells compared to Scrambled. A minimum of 200 cells from each of two biological replicates, for each treatment, was counted. All data are means ± SEM. Statistical significance was assessed by one-way ANOVA, followed by Tukey’s post-test. **p < 0.01.

To directly test the role, if any, of USP9X in NPs we altered its levels in the immortalized human NP cell line, ReNcell VM. To deplete USP9X in these cells, lentiviral vectors with doxycycline-inducible expression of shRNAs directed against *USP9X* were generated^16^. The lentiviral vector also expressed *EGFP*, which was used to identify and FACS purify, successfully transduced pools of cells, for subsequent experiments. Two independent shRNAs (2193 and 4774, indicating the position of the first base pair of the shRNA in the *USP9X* open reading frame) efficiently depleted USP9X and these cell lines were chosen for future experiments. Induction of a scrambled shRNA, as well as the addition of doxycycline, had no effect on USP9X protein levels (Fig. 1A). Partial loss of USP9X was evident 24 hours after doxycycline addition in 2193 and 4774 cells, and reached maximal levels by 72 hours (Fig. 1A). To examine the effect of USP9X depletion on ReNcell VM and determine the time of maximum effect, cells were analyzed using the xCELLigence system, which measures electrical impedance, and so is proportional to cell number, in real time. Analysis of two biological replicates revealed that the cell index of ReNcell VM cells expressing *USP9X* shRNAs plateaued approximately 20 hours after doxycycline addition (Fig. 1B). In 2193 and 4774 cells the decrease in cell index reached statistical significance (p < 0.05; analysis was obtained by plotting t-values of the two way ANOVA analysis versus time) at 34 hours and 28 hours, respectively. At 24 hours USP9X protein levels were not yet fully depleted (Fig. 1A). These data indicate that ReNcell VM NPs are particularly sensitive to reduced USP9X levels.

### USP9X depletion decreases the proliferation of ReNcell VM cells but does not result in morphological changes, apoptosis or differentiation

The xCELLigence cell index, which measures electrical impedance, does not distinguish between effects on cell proliferation, morphology or cell death. Therefore we examined the effect of *USP9X* knock-down on these functions. No changes in ReNcell VM morphology were observed but a clear reduction in the cellular density of USP9X depleted ReNcell VM could be distinguished at 72 hours after doxycycline treatment (Supplementary Fig. S1A). In addition, lower MTT levels were detected in USP9X depleted ReNcell VM after 48 and 72 hours doxycycline treatment consistent with decreased cell numbers (Supplementary Fig. S1B). Analysis of both Annexin V (Supplementary Fig. S2) and cleaved caspase 3 (Supplementary Fig. S3A) indicated there was no increase in apoptosis, after 12 and 72 hours doxycyline treatment, respectively. Immunoblot analysis failed to detect any change in the level of the astrocytic marker GFAP or neuronal marker βIII-tubulin, indicating that loss of USP9X did not induce the differentiation of ReNcell VM cells to post-mitotic cell fates (Supplementary Fig. S3B). Finally, to directly determine the effect on USP9X knockdown on cell proliferation an EdU pulse assay was performed to label cells undergoing DNA synthesis during S-phase. Significantly fewer EdU positive cells were detected in 2193 and 4774 cells treated with doxycycline for 72 hours compared to those expressing the scrambled shRNA (Fig. 1C,D). These data demonstrate that loss of USP9X decreases the proliferation of ReNcell VM cells but does not alter their morphology, cell death or differentiation.

### USP9X depletion results in G0/G1 cell cycle arrest

**Figure 2:**
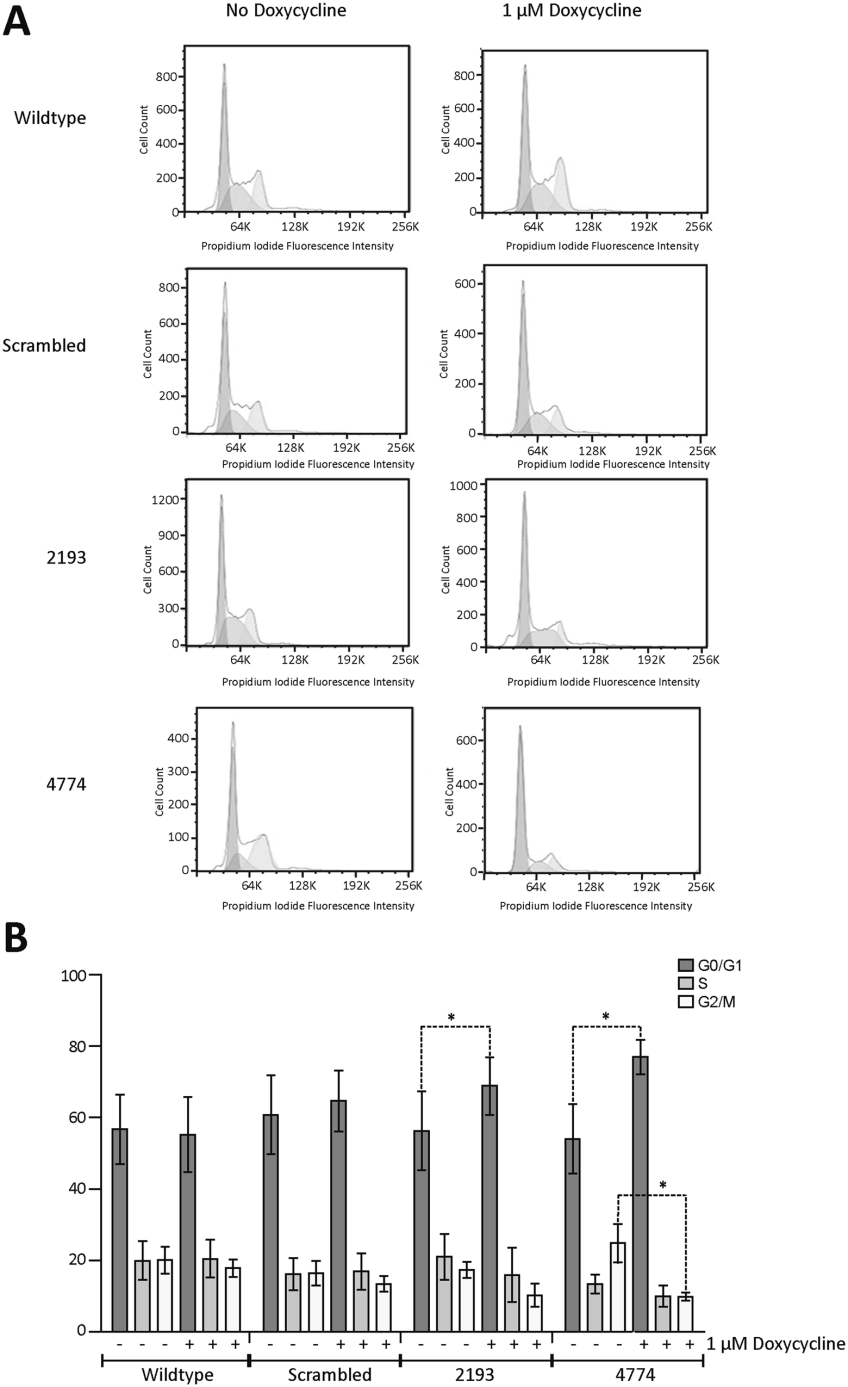
Loss of USP9X results in ReNcell VM cell accumulation in the G0/G1 phase of cell cycle. (**A**) Flow cytometric analysis of ReNcell VM cells after 72 hours of doxycycline treatment. Representative of three biological replicates. (**B**) Quantitation of percentage of cells in each cell cycle phase from three biological replicates. All data are shown as the means ± SEM. Statistical significance was assessed by the unpaired Student’s t test. *p < 0.05.

To investigate if USP9X depletion affected ReNcell VM cell cycle, flow cytometric profiling of cellular DNA content was conducted. Knock-down of USP9X resulted in an increase in cells at the G0/G1 stage of the cell cycle 72 hours after doxycycline treatment (Fig. 2A,B). Over three biological replicates the average increase in cells in G0/G1 following USP9X depletion was 12.6% ± 6.3 (p = 0.0134) for “2913” and 22.91% ± 8.9 (p = 0.0065) for “4774” cells. There was no statistically significant alteration in wildtype or scrambled shRNA cells exposed to doxycycline (Fig. 2B). Removal of doxycycline from the culture medium resulted in restored levels of USP9X and ReNcell VM proliferation (data not shown). These data indicated that USP9X is required for progression of ReNcell VM cells through G0/G1 and/or progression into S-phase. To investigate the molecular mechanism contributing to the accumulation of cells in G0/G1 phase we examined, by immunoblot, the levels of various proteins facilitating the G1/S phase transition. A decrease in the phosphorylated form of retinoblastoma protein (pp-Rb^S780^) was detected in USP9X depleted cells, but only after 72 hours of doxycycline treatment. No consistent changes in other proteins including Cyclin D1 or E2F1 were observed at any stage of the time course (Supplementary Fig. S4). As the xCELLigence cell index of USP9X depleted ReNcell VM cells plateaued at 24 hours this suggested that decreases in pp-Rb^S780^ were unlikely to be the mechanism initiating the G0/G1 arrest.

### USP9X depletion in ReNcell VM cells reduces mTORC1 signalling

**Figure 3:**
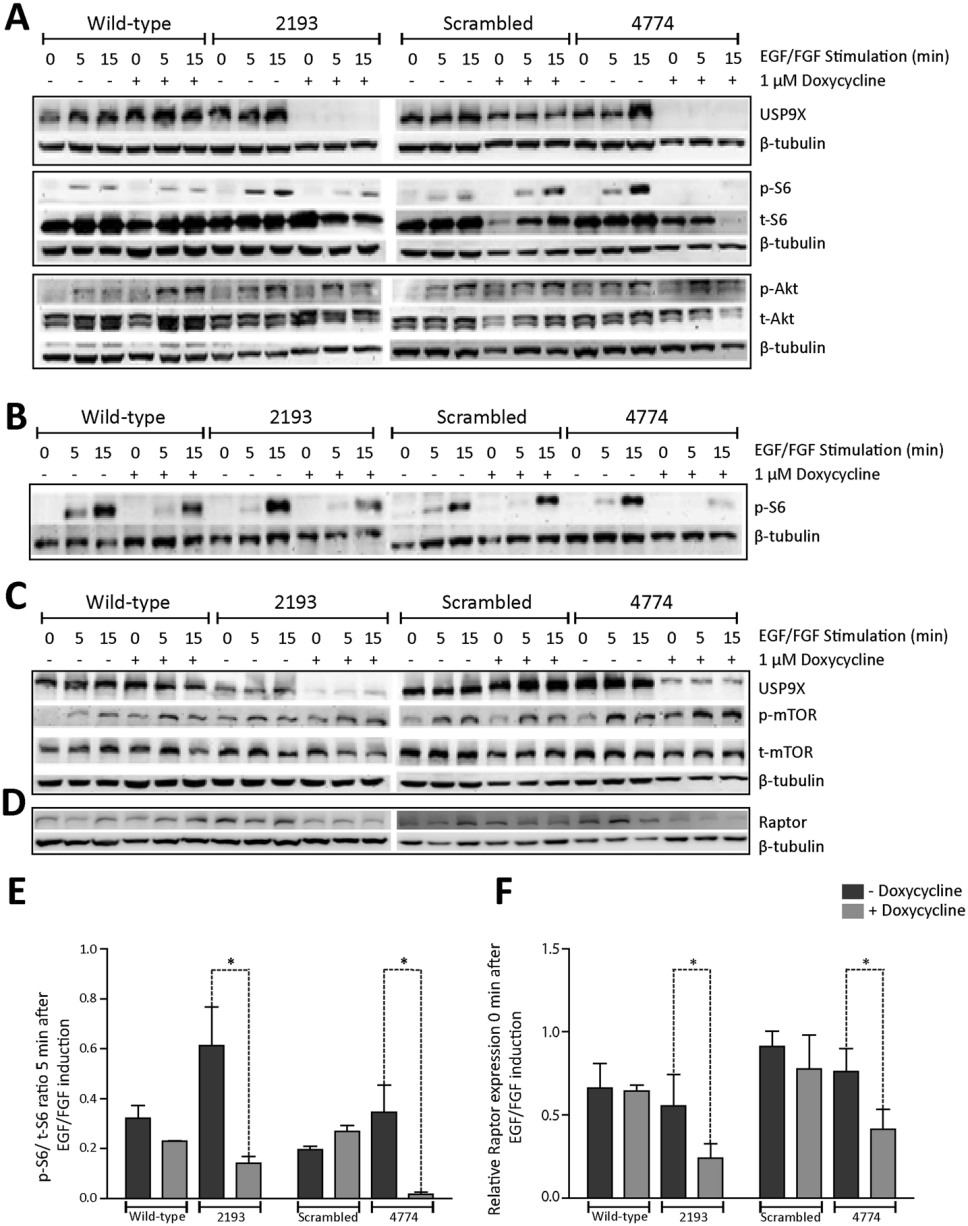
mTORC1 activity and RAPTOR levels in ReNcell VM cells are dependent on USP9X. (**A**) Immunoblot analysis of growth factor induction of mTORC1 and mTORC2 pathways in absence of USP9X. USP9X was depleted from 2193 and 4774 cells by 72 hours exposure to doxycycline before growth factors (EGF/FGF) were removed from ReNcell VM medium 24 hours prior to protein collection (0 minutes) and then 5 and 15 minutes after re-addition of EGF/FGF. Both pS6 and pAkt levels increased upon exposure to EGF/FGF (compare 0 to 5 and 15 min) indicating both mTORC1 and mTORC2 pathway activation, respectively. However, the extent of induction for pS6 was markedly reduced in the absence of USP9X (compare plus and minus Dox at 5 and 15 mins in 2193 and 4774). Total S6 and Total AKT were not affected by USP9X depletion. Representative of three biological repeats. (**B**) Diminished mTORC1 activation in ReNcell VM cells, as determined by pS6 levels, was evident after only 24 hours doxycycline treatment, consistent with the xCELLigence data. (**C**) Phospho and Total mTOR levels were unaffected by the absence of USP9X. Representative of two biological replicates. Full length blots presented in Supplementary Fig. S8. (**D**) RAPTOR protein levels were decreased in ReNcell VM cells with depleted USP9X (2193 and 4774 plus doxycycline) Representative of three biological replicates. (**E**) Densitometric quantitation, of immunoblots from two biological replicates, of initial pS6 induction compared to total S6 levels 5 mins after addition of EGF/FGF in the absence or presence of doxycycline. The treatment of cells with doxycycline significantly attenuated the level of pS6 in 2193 and 4774 cells only. (**F**) Densitometric quantitation of immunoblots, from three biological replicates, of RAPTOR protein, relative to β-tubulin control, at time 0 hour in the absence or presence of doxycycline. The treatment of cells with doxycycline significantly attenuated the level of RAPTOR in 2193 and 4774 cells only. All data are shown as the means ± SEM. Statistical significance was assessed by the unpaired Student’s t test. *p < 0.05.

It has recently been reported that USP9X interacts with the mTOR signalling pathway^15^, a major regulator of progression through the G1 phase of the cell cycle. mTOR exists in two complexes, mTORC1 and mTORC2, and the activity of each pathway is measured by the phosphorylation of their substrates, S6 and Akt, respectively^15, 17–19^. In the absence of growth factor stimulation, cells arrest in G0. However, re-addition of growth factors promptly activates mTOR signalling. Therefore we examined the activation of mTOR in ReNcell VM cells, by the re-addition of EGF and FGF, in the presence and absence of USP9X. ReNcell VM cells were exposed to doxycycline for 72 hours for maximal knock-down of USP9X, and a further 24 hours in the absence of EGF and FGF to cause G0 arrest. Upon the reintroduction of EGF and FGF there was a rapid increase in pS6 levels, dependent on the presence of USP9X (Fig. 3A,E). A failure to induce pS6, in the absence of USP9X, was observed in each of six biological replicates. There were no obvious or consistent alterations in pAKT, total S6 (Fig. 3A) or total AKT (data not shown) protein levels across the biological replicates. To determine if the decreased mTORC1 signalling occurred with similar kinetics to the observed cell index plateauing (Fig. 1B) upon USP9X depletion, we examined pS6 protein levels following 24 hours doxycycline treatment. A similar decrease in pS6 levels in the absence of USP9X was detected at this earlier time point (Fig. 3B) suggesting this may account for the plateauing cell index detected in the xCelligence assay.

**Figure 4:**
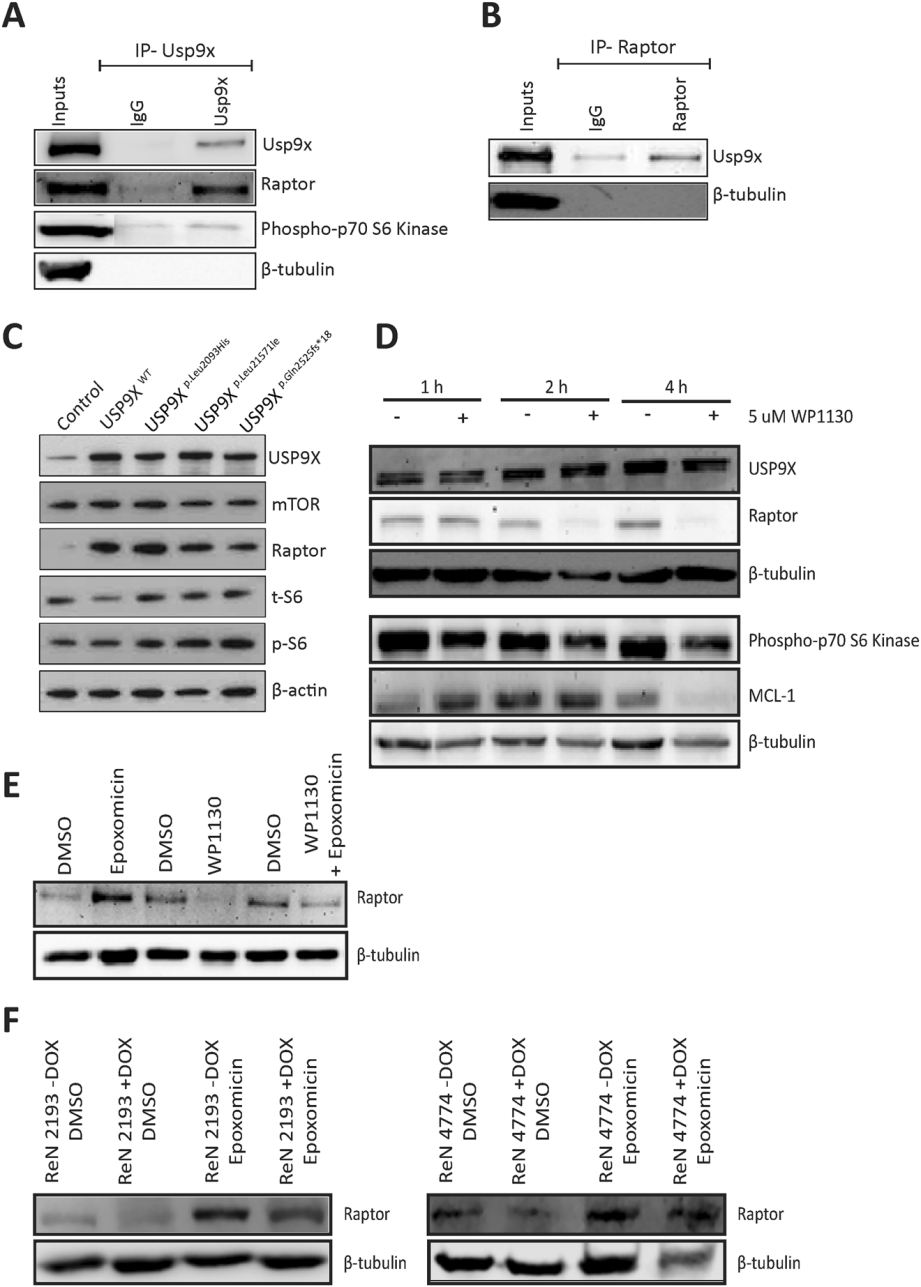
USP9X regulates RAPTOR levels and opposes its proteasomal degradation in neural progenitors. (**A**,**B**) Co-immunoprecipitation of endogenous Usp9x and Raptor from E14.5 mouse frontal cortex. (**A**) Anti-Usp9x antibodies precipitated Raptor protein. An interaction with pp70S6 kinase was also detected. (**B**) Anti-Raptor antibodies precipitated Usp9x protein. (**C**) USP9X over-expression increased RAPTOR protein levels. Proliferating HEK293T cells were transiently transfected with empty vector, full length USP9X (WT) or one of three USP9X variants, which do not affect enzymatic activity but are associated with human intellectual disability. Cell lysates were collected 24 hours later. Immunoblot analysis identified increased RAPTOR protein but mTOR and total S6 levels were unaffected. (**D**) Chemical inhibition of USP9X deubiquitylating activity leads to a rapid depletion of RAPTOR protein in neural progenitors. ReNcell VM cells were treated with 5 μM WP1130 and analysed by immunoblot. WP1130 does not deplete USP9X protein levels over 4 hours but results in almost complete loss of RAPTOR by 2 hours. pp70S6 kinase levels were not affected. Levels of another USP9X substrate MCL1 were depleted after 4 hours. (**E**) RAPTOR protein levels are regulated by proteasomal inhibition (epoxomicin) and inactivation of USP9X deubiquiytlating activity (WP1130) in ReNcell VM cells. Proteasomal inhibition following 4 hours exposure to 25 nM epoxomicin resulted in increased RAPTOR levels. Inhibition of USP9X DUB activity with 5 μM WP1130 for 4 hours resulted in decreased RAPTOR. Treatment of wild-type ReNcell VM cells for 4 hours with WP1130 followed by 4 hours of epoxomicin resulted in intermediate levels of RAPTOR. DMSO was used as the vehicle control for each experiment. Final concentrations of DMSO were – 0.0025% for epoxomicin; 0.00005% for WP1130; 0.00255% for WP1130 + Epoxomicin. (**F**) Epoxomicin treatment of 2193 and 4774 ReNcell VM cells resulted in increased RAPTOR protein levels. The addition of doxycycline (+DOX) resulted in intermediate levels of RAPTOR. Representative of two biological replicates.

Phosphorylation of S6 occurs at the end of the mTORC1-signalling cascade. We therefore examined the protein levels of upstream components of mTORC1, and mTORC2. The levels of total and p-mTOR (Fig. 3C and Supplementary Fig. S5A–C), phospho-p70S6 kinase (pp70S6K, data not shown) and RICTOR (Supplementary Fig. S5D), were not altered in the absence of USP9X following 72 hours of doxycycline treatment, however, a reduction in total RAPTOR levels was evident (Fig. 3D,F) and observed in three additional biological replicates (Figs 3F and 4F and Supplementary Fig. S6).

### USP9X binds and regulates RAPTOR levels

RAPTOR is the major scaffolding protein of the mTORC1 complex and also binds p70S6K^20, 21^. The level of RAPTOR protein is reportedly regulated by ubiquitylation^22, 23^ raising the possibility that USP9X maintains mTORC1 signalling by opposing RAPTOR degradation in the ubiquitin-proteasome system. If so, the levels of substrate, RAPTOR, should correlate with those of the deubiquitylating enzyme, USP9X. We’ve shown that loss of USP9X correlated with decreased levels of RAPTOR (Fig. 3D,F, Supplementary Fig. S6). To further investigate the relationship between these proteins, we determined if endogenous Usp9x and Raptor proteins interacted by performing immunoprecipitation on lysate from the frontal cortex of embryonic mouse brains, which are enriched for NPs. A clear interaction between endogenous Usp9x and Raptor proteins was detected, as was a weaker interaction with p70S6K in E14.5 tissue (Fig. 4A). Interestingly, no interaction between Usp9x and mTOR was detected under these conditions. In addition, immunoprecipitation using anti-RAPTOR antibodies enriched for Usp9x (Fig. 4B). Therefore USP9X and RAPTOR interact *in vivo*. Next, USP9X was transiently over-expressed in HEK293 cells (due to low transfection efficiency of ReNcell VM cells). Increased levels of USP9X resulted in increased RAPTOR but did not alter the level of other mTORC1 pathway components including mTOR or total S6 (Fig. 4C, Supplementary Figure S7). Over-expression of three USP9X mutations, which are associated with human intellectual disability but do not affect their deubiquitylating activity^13^, also increased RAPTOR levels (Fig. 4C).

The above loss- and gain-of-function approaches, in human ReNcell VM cells and HEK293T cells, respectively, identified a direct correlation between USP9X and RAPTOR protein levels but they do not indicate if USP9X’s deubiquitylating activity is required. Recently a small compound, WP1130, has been identified which inhibits USP9X DUB activity, but does not affect USP9X protein level^24^. In addition, WP1130 rapidly inhibits USP9X within hours, compared with days for RNA knock-down approaches and thereby circumvents possible induction of compensatory DUB expression^24^. The addition of WP1130 to proliferating ReNcell VM cells under normal culture conditions, in the presence of EGF and FGF, resulted in a rapid (2 hours) decrease in RAPTOR protein levels but had no effect on pp70S6K (Fig. 4D and Supplementary Fig. S7). As reported by others, WP1130 did not affect the level of USP9X protein, but did deplete another USP9X substrate, MCL1^25^ after 4 hours. Therefore, inhibition of USP9X’s deubiquitylating activity leads to a rapid decrease in RAPTOR protein in ReNcell VM cells.

### USP9X opposes proteasomal degradation of RAPTOR

The above data are consistent with USP9X opposing the degradation of RAPTOR in the ubiquitin-proteasome system. To directly examine this, we measured RAPTOR levels in ReNcell VM cells treated with the proteasome inhibitor, epoxomicin^26^. Epoxomicin treatment for 4 hours increased RAPTOR levels (Fig. 4E lane 2) suggesting it is subject to proteasomal degradation in ReNcell VM cells. As shown previously, inhibition of USP9X deubiquitylating activity by WP1130 resulted in depleted RAPTOR levels (Fig. 4E lane 4). The combination of epoxomicin and WP1130 resulted in intermediate levels of Raptor protein compared with epoxomicin or WP1130 alone (Fig. 4E, compare lane 6 with 2 and 4). These data are consistent with USP9X’s deubiquityling activity opposing degradation of RAPTOR by the proteasome. However, as WP1130 activity inhibits deubiquitylating enzymes other than USP9X^27^ proteasomal inhibition experiments were repeated in the 2193 and 4774 ReNcell VM lines to validate a specific role for USP9X in the inhibition of RAPTOR degradation. As for WP1130, doxycycline knockdown of USP9X produced similar results (Fig. 4F, Supplementary Fig. S8). Furthermore, qRT-PCR analysis indicated that loss of USP9X did not affect Raptor mRNA levels (Supplementary Fig. S9) consistent with post-translational regulation.

### USP9X null neurospheres have reduced neural progenitor proliferation but not stem cell self-renewal

**Figure 5:**
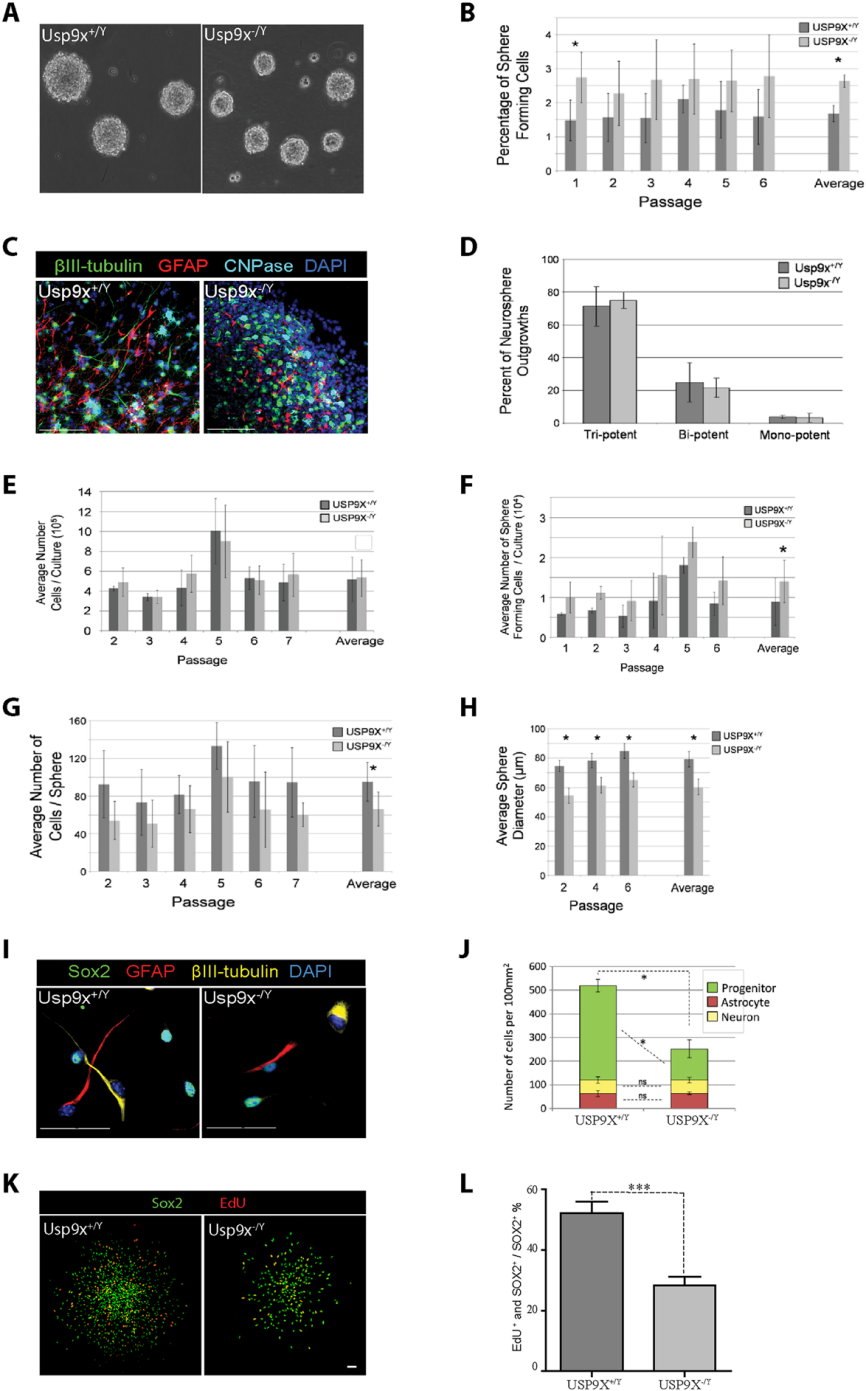
Loss of *Usp9x* promotes stem-cell self-renewal and loss of bulk NP proliferation. Neurosphere cultures were derived from the cortices of E18.5 *Usp9x* ^+/Y^ (n = 4) and *Usp9x* ^−/Y^ (n = 5) mice. (**A**) Representative phase contrast images *Usp9x* ^+/Y^ and *Usp9x* ^−/Y^ neurospheres. (**B**) *Usp9x* ^−/Y^ cultures contain a higher percentage of sphere-forming cells. The average percentage of cells from dissociated neurospheres, that re-form spheres at very low density, was derived over several passages. (**C**,**D**) Loss of *Usp9x* does not affect the multipotency of sphere-forming cells. Whole neurospheres (passage 2, n = 3 for *Usp9x* ^−/Y^ and *Usp9x* ^−/Y^) were plated at low density and outgrowths stained and scored after 6 days. (**C**) Representative image of cultures labelled for cell type marker proteins GFAP: astrocytes; βIII-tubulin: neurons and CNPase: oliogodendrocytes. (**D**) The percentage of neurosphere outgrowths displaying tri - bi and mono-potency scored and compared by Student t-test (not statistically different). (**E**) *Usp9x* ^+/Y^ and *Usp9x* ^−/Y^ cultures contain equivalent number of cells at each passage. (**F**) *Usp9x* ^−/*y*^ cultures contain increased numbers of sphere forming cells. Total sphere-forming cells calculated across several passages. (**G**,**H**) *Usp9x* ^−/Y^ neurospheres are smaller. Mean diameter and number of cells per neurosphere at each passage. (**I**,**J**) Loss of *Usp9x* decreases NP number. Neurosphere cultures (passage 2, n = 3 for *Usp9x* ^−/Y^ and *Usp9x* ^−/Y^) were dissociated and plated at very low density onto poly-l-lysine coated plates in media containing EGF and cultures fixed 24 hours later. (**I**) Representative image of immunofluorescently stained cultures; cells labelled for cell markers; Sox2: NP; GFAP: astrocytes; βIII-tubulin: neurons. Cell nuclei counterstained with DAPI. (**J**) Cell counts used to quantify numbers of cell types present in cultures. (**K**,**L**) Loss of *Usp9x* inhibits NP cell proliferation. (**K**) Representative images of neurospheres allowed to attach for 2 hours followed by a 6 hour EdU pulse and immunostained for the NP marker Sox2 (Green) and EdU (Red). (**L**) Lower percentage of *Usp9x* ^−/*Y*^ NPs (Sox2-positive) were labelled with EdU indicating diminished NP proliferation. Scale bar = 50μm. All data are shown as the means ± SEM. Statistical significance was assessed by the unpaired Student’s t test.*p < 0.05; ***p < 0.001.

The mTORC1 pathway is well known to regulate murine NP proliferation. Inactivation of mTORC1 signalling in NPs *in vivo*, by conditional Nestin-*cre* mediated deletion of the *Raptor* gene in mouse brains, resulted in reduced NP proliferation. When mTORC1 signalling is inhibited in NSC/NPs cultured *ex*-*vivo* as neurospheres, the reduced NP proliferation manifested as a reduction of neurosphere size^8^. We therefore performed neurosphering assays on *Usp9x* ^−/*Y*^ NSC/NPs derived from late embryonic stage mouse brains following Nestin-*cre* deletion of *Usp9x* ^12^. Neurospheres contain a heterogeneous population of NSC, NP and differentiated neural cells^28^. Upon single cell dissociation and re-culture at very low density, to exclude cell aggregation, only cells with stem cell-like properties reform neurospheres^29–32^. These spherogenic neural stem cells typically represent only ~1–5% of the cells derived from neurospheres^28, 30, 32^. In contrast NPs have very limited capacity to reform spheres over multiple passages. We applied the well-established serial passage sphere-forming assay to identify the effect, if any, of Usp9x loss on the long-term self-renewal of the spehrogenic NSC population. In this assay, a set number of single NPs are plated at very low density following each passage (1 × 10^4^ cells/ml) and the number of spheres that form in those cultures then counted. Over several passages, *Usp9x* ^−/Y^ cultures consistently contained significantly more neurosphere forming cells (2. 6% compared to 1.7%; p < 0.05; Fig. 5B). Next we asked if the loss of Usp9x had any effect on the multipotency of the NSC/NPs (Fig. 5C,D). As each sphere is derived from a single cell, the cell types a sphere can produce reflects the multipotency of the original sphere-forming cell^28, 32^. Spheres were plated at very low density and allowed to differentiate before identifying the three basic neural lineages that are produced from embryonic NSCs, namely, neurons, astrocytes and oligodendrocytes (Fig. 5C). We scored individual spheres for the presence of all three, two or one lineages as tri-, bi and mono-potent respectively. The majority of spheres (70–73%) were tri-potent, and there were no significant differences between *Usp9x* ^+/Y^ and *Usp9x* ^−/Y^ cultures, suggesting loss of Usp9x had no effect on the multipotency of the sphere-forming stem cells (Fig. 5D). Given the increase in the number of spheres in *Usp9x* ^−/Y^ cultures, we asked if this resulted into an increase in the absolute number of cells that were produced during culture, however, both *Usp9x* ^+/Y^ and *Usp9x* ^−/Y^ cultures produced equivalent cell numbers across several passages (Fig. 5E). This finding had two implication; firstly that the increased percentage of sphere forming cells in *Usp9x* ^−/Y^ cultures translated into increases in the absolute number of sphere forming cells in the cultures (Fig. 5F); and secondly, that individual neurospheres in *Usp9x* ^−/Y^ cultures must contain fewer cells. Indeed, on average, neurospheres in *Usp9x* ^−/Y^ cultures displayed modest but significant reductions in the number of cells per sphere (30% reduction; ~95 compared to ~66 cells/sphere), which was also reflected in reductions in the average sphere diameter (24% reduction; ~79 compared to ~60 μm/sphere) at the time of passaging (Fig. 5A,G,H). Therefore, while loss of Usp9x promoted the self-renewal and expansion of the sphere-forming population, the bulk growth of the sphere after formation was compromised. To investigate if this loss of bulk growth could be attributed to altered differentiation behaviour, we used immunofluorescence to reveal the expression of cell-type specific marker proteins to identifying cell types present in the spheres. As it is technically difficult to resolve in the 3D environment of a neurosphere, we dissociated the neurosphere cultures into single cells and plated them onto coverslips. Following attachment overnight and 24 hours growth, cells were fixed and stained for marker proteins of NPs (Sox2), neurons (βIII-tubulin) and astrocytes (GFAP) and nuclei counterstained with DAPI (Fig. 5I). Using this labelling regime, greater than 99% of all cells were labelled (data not shown). At this time, there was already a 51% reduction in total cell numbers (Fig. 5J). This reduction was solely due to a loss of Sox2-positive NPs (67% decrease) with the numbers of astrocytes and neurons being equivalent. To directly test if the absence of *Usp9x* affected NP proliferation, neurospheres were exposed to a 6 hour EdU pulse followed by cell counts (Fig. 5K,L) or flow cytometry (Supplementary Fig. S9). As was observed for ReNcell VM cells, significantly fewer Sox2-positive NPs were proliferating (EdU-positive) in the Usp9x-deleted neurospheres.

In aggregate these data strongly suggest the absence of Usp9x in neurospheres promotes the self-renewal of a rare population of sphere-forming cells that displays the properties consistent with that of NSCs, whilst simultaneously inhibiting the proliferative capacity of the bulk transiently amplifying NP population. This phenotype, of smaller neuropsheres and reduced NP proliferation but not their self-renewal, is shared with the central features of both Raptor null or rapamycin treated neurospheres^8^ and together suggests that Usp9x may also be required to regulate mTORC1 function in NPs in neurospheres.

### USP9X regulates mTORC1 activity *in vivo*

**Figure 6:**
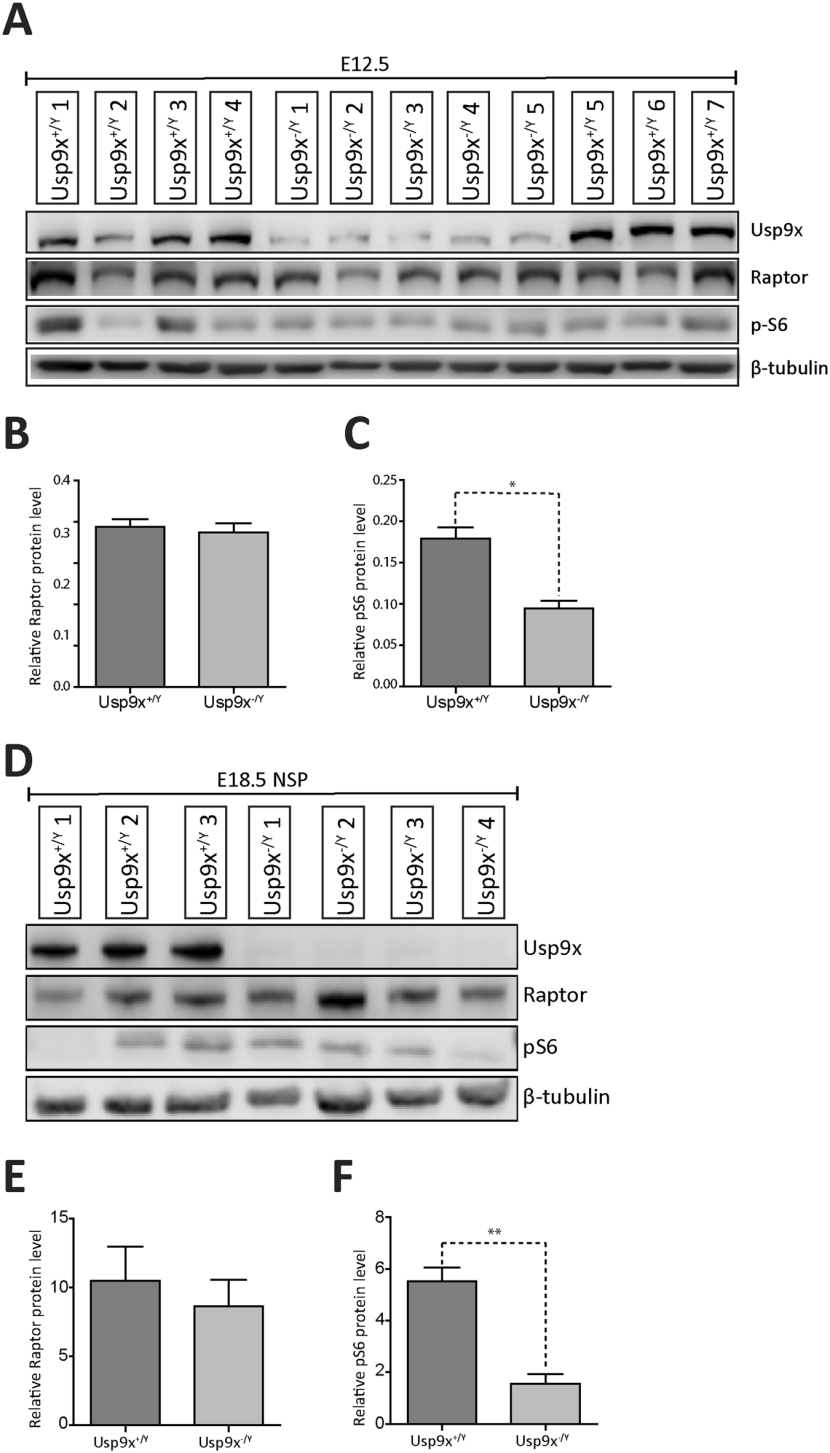
USP9X binds RAPTOR and regulates mTORC1 activity *in vivo*. (**A**) Immunoblot analysis of Usp9x, Raptor and pS6 protein from E12.5 *Usp9x* ^+/*Y*^ (n = 7) and *Usp9x* ^−/*Y*^ (n = 4) mouse forebrains. (**B**,**C**) Densitometric analysis of (A) revealed no change in Raptor (**B**) but significantly lower levels of pS6 (**C**) protein levels in *Usp9x* ^−/*y*^ brains. (**D**) Immunoblot analysis of Usp9x, Raptor and pS6 protein from E18.5 derived neurospheres (3 *Usp9x* ^+/*Y*^ versus 4 *Usp9x* ^−/*Y*^). No difference was observed in Raptor protein levels (**E**) but pS6 levels (**F**) were significantly decreased in Usp9x^−/Y^ neurospheres. All data are shown as the means ± SEM. Statistical significance was assessed by the unpaired Student’s t test. *p < 0.05; **p < 0.01.

Next the functional consequence of Usp9x depletion on Raptor levels and mTORC1 signalling *in vivo* during embryonic brain development was determined by immunoblot. Significantly lower levels of pS6 protein were detected in *Usp9x* ^−/Y^ brains at E12.5, however in contrast to cultured human ReNcell VM and HEK293 cells no effect was observed on Raptor (Fig. 6C–E). Similar analysis on neurospheres derived from E18.5 brains confirmed a dependence of pS6 levels on Usp9x, but not Raptor (Fig. 6F–H). These data support a role for USP9X in the maintenance of mTORC1 pathway signalling in both mouse and human NPs.

## Discussion

*USP9X* is highly expressed in, and regulates the function of, NPs both *in vivo* and *in vitro* ^11, 12, 33^. Previously, increased expression of *Usp9x* was shown to regulate NP self-renewal by promoting apical-basal cell polarity and altering the architecture of NP cultures derived from mouse embryonic stem cells^11^. Here we show USP9X is also required to maintain proliferation of the human NP line ReNcell VM, in a cell autonomous manner, and this is probably mediated via facilitating mTORC1 pathway signalling.

Inducible knock-down of USP9X demonstrated that ReNcell VM cells are exceptionally sensitive to USP9X depletion. Within 24 hours of initiating USP9X knock-down, proliferation of the ReNcell VM cells halted (Fig. 1) despite the presence of mitogenic quantities of EGF and FGF. Considering that USP9X protein levels were not completely depleted at this time (in some experiments USP9X was only 50% depleted) and, that ReNcell VM have a cell cycle time of approximately 30 hours, this indicates that even partial depletion of USP9X results in a rapid and comprehensive arrest of proliferation. Although apoptosis can quickly alter cell numbers, and the anti-apoptopic protein MCL1 is a USP9X substrate in some cellular contexts^25^, no alterations in Annexin V or cleaved caspase 3 levels were detected, indicating no increase in apoptosis (Supplementary Figs S2 and S3). Nor did USP9X depletion promote the differentiation of ReNcells VM to post-mitotic neuronal or glial lineages (Supplementary Fig. S3). Instead the NPs accumulated in the G0/G1 phase of the cell cycle (Fig. 2). This rapid response was not due to alterations in G1/S check-point proteins, although a delayed decrease in pRb levels was observed.

The mTOR pathway regulates progression through the G0/G1 stage of the cell cycle^2^ and Usp9x influences mTORC1 and mTORC2 pathway activity in the C2C12 mouse myoblast cell line^15^. Here we show that USP9X is required for mTORC1, but not mTORC2, activity in ReNcell VM human NPs. The depletion of USP9X levels, loss of mTORC1 signalling, as indicated by failure to induce p-S6, and ReNcell VM cell-cycle arrest, all occurred with very similar kinetics strongly suggesting these NPs rely on USP9X-dependent mTORC1 activity for proliferation.

Post-translational modification of mTOR proteins, including by ubiquitylation, allow the mTOR pathway to respond rapidly to changing extra-cellular signals. Both p-mTOR and RAPTOR are targeted for proteasomal degradation by poly-ubiquitylation^23, 34^. Here we present several lines of evidence identifying the canonical mTORC1 scaffold protein, RAPTOR, as a probable USP9X substrate in NP/NSCs. Firstly, we show that endogenous USP9X and RAPTOR proteins co-immunoprecipitate from mouse embryonic brain tissue enriched for NPs (Fig. 6A,B). USP9X did not interact with mTOR under the same conditions. The much weaker interaction detected between USP9X and pp70S6K (Fig. 6A) may be direct or indirect via RAPTOR, which binds pp70S6K to recruit it to the mTORC1 complex^21^. The absence of any alteration in pp70S6K levels following the inhibition of USP9X activity (Fig. 4B), suggests USP9X does not directly regulate phospho-p70S6K.

Second, inducible USP9X-specific shRNA-mediated knock-down (Fig. 3C,E, Supplementary Fig. S6), and transient over-expression of full length USP9X (Fig. 4A) resulted in a concomitant decrease and increase in RAPTOR protein, respectively. Use of the chemical WP1130, which rapidly inhibits USP9X’s deubiquitlyating activity but does not deplete USP9X protein levels, confirmed a clear and rapid loss of RAPTOR (Fig. 4C). The near complete depletion of RAPTOR protein within 2 hours strongly suggests that USP9X regulation occurs at the post-translational level. This is supported by the observation that the loss of MCL1, a bona fide USP9X substrate, occurred only after 4 hours in the same experiment (Fig. 4C). However, as WP1130 inhibition is not restricted to USP9X, we cannot rule out the possibility that other DUBs, in addition to USP9X, might also regulate RAPTOR stability. To address this we analysed ReNcell VM cells with inducible USP9X-specific shRNA-mediated knockdown and these produced similar results to WP1130 (Fig. 4D) suggesting USP9X plays a significant, if not exclusive, role in maintaining RAPTOR levels in NPs.

An obvious hypothesis is that USP9X’s deubiquitylating activity opposes RAPTOR’s degradation at the proteasome. Data presented (Fig. 4C,D) confirmed that inhibition of the proteasome increased RAPTOR protein levels in ReNcell VM cells as observed in HEK293T cells^23^, and that inhibiting the proteasome, in the absence of USP9X activity partially rescued RAPTOR levels. USP9X is the first deubiquitylating enzyme shown to stabilize, components of the mTOR pathway namely, RAPTOR. Although another deubiquitylating enzyme, UCH-L1 has been shown to impact upon mTORC1 signalling it does so indirectly by affecting assembly of the mTORC1 complex^23^. Unlike USP9X, UCH-L1 has no effect on the protein levels of mTOR complex components including RAPTOR.

USP9X’s regulation of mTOR signalling appears to be cell context specific. Recently it was shown in C2C12 muscle myoblasts that epitope-tagged USP9X weakly associated with mTOR and both RAPTOR and RICTOR in HEK293T cells and its depletion in C2C12 myoblasts accelerated their differentiation to myotubes^15^. In contrast to our results in NPs, Usp9x depletion from C2C12 cells led to increased mTORC1 activity, in response to growth factor stimulation. Interestingly, while Usp9x depletion in C2C12 cells altered the downstream mTORC1 and mTORC2 effectors, pS6 and pAKT, respectively, no alteration in the levels of the upstream proteins mTOR or RAPTOR, were detected. Therefore in C2C12 myoblasts, the regulation of mTOR signalling by Usp9x may be indirect. Cell context specific roles of USP9X have also been observed in other systems^25, 35, 36^. Although Usp9x has been identified as a potential regulator of stem cell function^37^, decreasing Usp9x levels does not overtly diminish the proliferation of other progenitor/stem cells including, embryonic stem cells^38^, T-cell progenitors^39^ or pancreatic progenitors^36^. In contrast, the data presented here indicates that both cultured human NPs and, those in mouse neurospheres, are particularly sensitive to USP9X levels.

mTOR signalling is a major regulator of NP function in both development and disease^4^. Our data establish that USP9X facilitates mTORC1 signalling in NPs in both cultured human NPs, neurospheres and the developing mouse brain. Our data also reveals the phenotype of Usp9x null neurospheres is very similar to that reported for rapamycin treated and Raptor depleted neurospheres^8^ and is therefore consistent with Usp9x stabilisation of mTORC1 signalling in mouse NPs as well. Loss of either Usp9x or Raptor resulted in smaller neurospheres but did not affect their capacity for serial passage indicating they affected proliferation of NPs without inhibiting the self-renewal capacity of the sphere-initiating NSC. Likewise rapamycin treatment of neurosphere cultures resulted in reduced NP proliferation (and reduced neurosphere size) without affecting the stem cell state^9^, and rapamycin delivery to the adult sub-ventricular zone neurogenic niche specifically depleted the transiently amplifying NPs but not the stem cell population^40^. These data are consistent with retention of transiently amplifying NPs in the G0 stage of the cell cycle, a phenomenon reported for adult neural stem cells exposed to rapamycin^40^. However, we did not detect a significant decrease in Raptor protein in Usp9x-null neurospheres, nor embryonic brain as assessed by immunoblot (Fig. 6C,D,F,G) like that observed in the human ReNcell VM neural progenitors. This may be due to the presence of a compensatory mechanism in the increasingly complex cellular composition of embryonic neural development over time or, the non-synchronised nature of cycling NPs or the presence of mitogens, as initial experiments on asynchronously growing ReNcell VM cells in the presence of mitogens also failed to detect significantly altered pS6 levels in, at least by immunoblot (data not shown). It is also possible that USP9X interacts with different mTORC1 components in human and mouse neural progenitors.

Finally, we observed that the loss of *Usp9x* resulted in more neurosphere-initiating stem cells at each passage, despite fewer proliferating NPs. This suggests that mTORC1 is not the only pathway regulated by USP9X in NPs, as inhibition of RAPTOR function in neurospheres did not produce a similar increase in sphere-forming cells. In addition, in embryonic stem cell-derived NPs *in–vitro*, a moderate increase in Usp9x levels promotes NP self-renewal^11^. Indeed USP9X regulates components of multiple pathways important to NP fate including the Notch, Wnt and TGF-β pathway^10^ and the final outcome is very much dependent on the type of NP. Consistent with a context-specific role for USP9X in the regulation of NP self-renewal we recently reported that loss of Usp9x has a differential effect on quiescent versus proliferating NSCs in the postnatal mouse hippocampal subgranular zone^33^. It is possible that Usp9x promotes the proliferation of intermediate progenitor cells at the expense of NSC self-renewal in the postnatal hippocampus. The differential dependence of NPs and sphere-forming NSCs on Usp9x may reflect a similar molecular mechanism. As Usp9x regulates brain development in mice^12^ and is associated with several human neurodevelopmental disorders^13, 41^ the precise role of Usp9x in NPs and stem cells warrants further investigation.

## Materials and Methods

### Generation of inducible Usp9x knock-down ReNcells VM lines

The human neural stem cell line ReNcells VM was obtained from Millipore (Darmstadt, Germany) and maintained as described^42^. To generate ReNcells VM with doxycycline-inducible knock-down of USP9X, cells were transduced with a lentiviral vector^43^ containing either a “scrambled” shRNA sequence: 5′ACTACCGTTGTTATAGGTGTTCAAGAGACACCTA TAACAACGGTAGT3′ with no homology to any known gene, or shRNA targeting two independent USP9X sequences “2193” 5′GCTTGATCCTTCCCTGTTAAC3′ (the first base pair is 2193 in the human USP9X sequence) or “4774” 5′GCCATAGAAGGCACAGGTAGT3′. Successfully transduced pools of ReNcells VM were isolated by FACS based on constitutive EGFP expression^43^. These transduced pools were used in subsequent experiments. Induction of shRNA expression was achieved by supplementing the media with 1 μM doxycycline.

### Functional analysis of ReNcells VM

#### xCELLigence Assays

The xCELLigence system for real-time cell viability monitoring was used as described^44^. Briefly, ReNcell VM cells were plated at 5 × 10^3^ per well and 1 μM doxycycline added the following day. The plate was analysed by xCELLigence (Roche, Basel, Switzerland) in real time over 5 days of doxycycline treatment.

#### MTS Assay

The MTS assays were performed according to the manufacturer’s instructions (Promega, AUS).

#### Annexin V Apoptosis Assay

Cells were seeded at 2 × 10^5^ cell/ml and treated with 1 μM doxycycline 12 hours later. After 12 hours treatment, cells were harvested by centrifugation at room temperature for 5 mins at 0.4 rcf and subjected to Annexin V assay as per the manufacturer’s instructions (Trevigen, Gaithersberg, USA).

#### Cell Cycle Analysis

Cells were seeded at 2 × 10^5^ cell/ml and treated with 1 μM doxycycline 12 hours later. Cells were in log phase at time of harvest. Cells were washed with HBSS and centrifuged for 5 mins at 0.3 rcf before suspension in 1 ml HBSS and fixation at 4 °C for 1 hour in 100% cold ethanol. Cells were stained with Propidium Iodide staining solution (3.8 mM Sodium Citrate, 50 μg/ml PI in DPBS) and 50 μl of RNase A stock solution (10 μg/ml RNase A in DPBS) was added. Tubes were stored at 4 °C until Flow cytometry analysis using a Becton Dickinson FACSAria.

### Molecular analyses

#### Immunoblotting

Cells were lysed with RIPA lysis buffer (150 mM NaCl, 1.0% Triton X-100, 0.5% sodium deoxycholate, 0.1% sodium dodecyl sulphate, 50 mM Tris, pH 8.0) supplemented with 100 μl/mL protease inhibitor. Cell lysate was then processed for immunoblotting and visualised with ECL substrate as described^12^. The following antibodies have been used: **Primary Antibodies**: Cell Signalling Technologies (Danvers, MA)*:* Rabbit-Cyclin D (1/1000), Mouse- Cyclin E (HE12) (1/1000), Mouse- Cyclin A (BF683) (1/2000), Rabbit- Caspase-3 (1/1000), Rabbit Cleaved Caspase-3 (Asp175) (1/1000), Rabbit p-S6S235/236 (1/800), Rabbit Total S6 (1/2000), Mouse IgG1 mTOR (1/1000), Rabbit p-mTORS2448 (1/800), Rabbit E2F1 (1/1000), Rabbit pp-RbS780 (1/1000), Rabbit Rb (1/2000), Rabbit Rictor (1/800), Rabbit Raptor (1/800), Rabbit p-AktS473 (1/1000), Rabbit t-Akt (1/2000), Rabbit p-p70S6K (1/500), Rabbit β-Tubulin (1/2000). Santa Cruz (Dallas, TX)*:* Rabbit-poly MCL1 (1/500). Purified Rabbit N-terminal^45^ (1/2000). Bethyl Labs (Montgomery, TX): Rabbit USP9x C terminal (1/2000) Sigma Aldrich (St Louis, MO): Mouse β-Tubulin (1/2000), Trevigen (Gaithersberg, USA)*:* Rabbit GAPDH (1/2000). **Secondary Antibodies**: Life Technologies (Mulgrave, AUS): Rabbit HRP (1/5000), Mouse HRP (1/5000). Millipore (Darmstadt, Germany)*:* Rabbit HRP (1/5000), Mouse HRP (1/5000).

#### Immunoprecipitation

Protein lysate was isolated from the frontal cortex of mid-gestation embryonic mouse brain to enrich for neural progenitors. The isolated tissues were lysed by sonication in the presence of the IP lysis buffer – 20 mM HEPES pH 7.8, 400 mM KCl, 5 mM EDTA, 0.4% NP40, 10% glycerol, 1 mM DTT, protease and phosphatase inhibitor cocktails (Cell Signalling, Danvers MA). Immunoprecipitation was conducted using Pierce co-immunoprecipitation kit (Thermo Scientific, Rockford, IL) according to manufacturer’s instructions. Immunoprecipitated proteins and total protein extracts were then subjected to immunoblot analysis.

#### Over-expression of USP9X

Transient expression of wild-type USP9X, and variant forms associated with intellectual disability, in HEK293T cells was as previously described^13^. Briefly, cells were transfected using Lipofectamine 2000 reagent as per manufactures instructions (Life Technologies). Cell lysates were collected after 24 hours and subjected to immunoblot analysis.

#### Neurosphere Culture

Isolation and culture of NPs from the E18 cortex was as previously described^28, 46^. All neurosphere-based assays were conducted in at least biological triplicate, with each replicate representing a neurosphere culture derived from individual Usp9x^+/Y^ or Usp9x^−/Y^ embryos. Averages of the replicates results are reported. Sphere forming assays were initiated by dissociating neurospheres and isolating single cells by passing dissociated cells through a 0.75 µm cell filter (BD Biosciences, San Jose, CA). Single cells were replated at very low density (1 × 10^4^ cells/mL) and cultured for 6 days. The number of spheres was calculated, and subsequently dissociated again to generate single cells for sphere forming assays. Number of cells per sphere was derived using total cell counts and total sphere number counts at each passage. At least 100 spheres were imaged per replicate and analysed using ImageJ to determine average sphere diameters. For the identification of cell types, dissociated single cells from neurospheres were plated onto poly-l-lysine (Sigma, St Louis, MO) coated coverslips (Menzel-glasser, Thermo Fisher Scientific) at 1 × 10^4^/cm^2^ in the presence of EGF and cultured for 24 hours at which point cells were fixed for immunofluorescent staining. The number of neuronal + astrocytes + progenitor cells was counted. Oligodendrocyte differentiation was negligible (<1%) and not included. At least 200 cells were counted per replicate (total n: *Usp9x* ^+/Y^ = 1299; *Usp9x* ^−/Y^ = 756). For analysis of multipotency, single cells derived from neurosphere dissociation and filtration were plated at very low density. Following 6 days culture, whole neurospheres were plated onto poly-l-lysine coated coverslips at low density (30 spheres/35 mm well) and cultured for a further 6 days. Neurosphere outgrowths were fixed and immunofluorescently stained for the presence of terminally differentiated cells namely neurons, astrocytes and oligodendrocytes. Neurosphere outgrowths were scored as Uni-, bi and tri-potent based on the presence of 1, 2 or all three cell types in the outgrowths, respectively. At least 20 outgrowths were scored per replicate (total n: *Usp9x* ^+/Y^ = 81; *Usp9x* ^−/Y^ = 68). All data points represent the average of replicate means and error bars represent standard deviation of replicate means. Statistical significance derived using Student’s 2-tailed t-test assuming equal variance and set to p < 0.05.

#### Immunofluorescence

Cultured cells were fixed with 4% PFA for 15 minutes at room temperature (RT) and block-permeablised using PBST-10% Normal Donkey Serum (NDS) for 1 hour at RT. Primary and secondary antibodies were incubated in 3%NDS overnight (O/N) at 4 °C and 1 hour at RT respectively at the following dilutions; mouse anti-CNPAse, (1:2000; Chemicon, Germany), rabbit anti-GFAP, goat anti-GFAP, mouse anti-βIII-tubulin, rabbit anti-βIII-tubulin (all at 1:300; Sigma-Aldrich), rabbit anti-Pax6 (1:200; Chemicon, Germany), donkey anti-sheep Alexafluor555, donkey anti-rabbit Alexafluor488/555/647 and donkey anti-mouse Alexafluor488/555/647 (all 1:1000; Invitrogen), donkey anti-chickenCy3 (Jackson Laboratories, Bar Harbour, ME). Cells were counterstained with DAPI and mounted with Slow-fade mounting media (Invitrogen). Non-specific staining was controlled by using secondary-only controls. Fluorescence was viewed using the Axioplan2 microscope (Carl Zeiss, Jena, Germany) fitted with an HBO 100 lamp (Carl Zeiss, Jena, Germany). Images were captured using an Axiocam Mrm camera and Axio Vs40 v4.5.0.0 software (Axiovision, Carl Zeiss, Jena, Germany).

#### Animal Use

This study was performed with the approval of both, the Griffith University Animal Ethics Committee under the guidelines of the National Health and Medical Research Council of Australia and the Australian Commonwealth Office of the Gene Technology Regulator as well as under regulations of the South Australian Animal Welfare Act 1986, and in strict accordance with the Australian Code of Practice for the Care of Animals for Scientific Purposes, 2004. The study was approved by the Women’s and Children’s Health Network (WCHN) Animal Ethics Committee (Approval Number: 750/06/2011 and 888/06/13). All experiments were carried out in accordance with the approved guidelines.

## Electronic supplementary material

Supplementary Information
