# Supplementary material for: USP9X deubiquitylating enzyme maintains RAPTOR protein levels, mTORC1 signalling and proliferation in neural progenitors
Source: Sci Rep. 2017 Mar 24;7:391. doi: 10.1038/s41598-017-00149-0 (PMC5427856; doi:10.1038/s41598-017-00149-0)
Supplement: Supplementary file 1 — Supplementary Information [file 41598_2017_149_MOESM1_ESM.pdf]

Title: USP9X deubiquitylating enzyme maintains RAPTOR protein levels, mTORC1 signalling and proliferation in neural progenitors.

Authors: Caitlin R. Bridges[1^], Men Chee Tan[1^], Susitha Premarathne[1^], Devathri Nanayakkara[1], Bernadette Bellette[1], Dusan Zencak[1], Deepti Domingo[3], Jozef Gecz[2,3], Mariyam Murtaza[1], Lachlan A. Jolly[2*], and Stephen A. Wood[1*]

SUPPLEMENTARY FIGURES

Supplementary Figure Legends

Supplementary Figure 1. (A) Deletion of USP9X reduces ReNcell VM culture density but does not overtly affect cell morphology. USP9X was depleted in ReNcell VM cells by 72 hours culture in presence of 1μM doxycycline. Bright field microscopy of Wildtype (A, A') Scrambled (B, B') 2193 'USP9x shRNA' (C,C') and 4774 'USP9x shRNA' (D,D') revealed no alteration in morphology but a reduction in cell density in 2193 (C') and 4774 (D') cultures with no pronounced effect on Wildtype (A') or Scrambled (B') cultures. **(B)** MTT assay analysis of RenCell VM cells after 48 hours and 72 hours treatment with 1 μM doxycycline showing reduction in USP9X knock-down cells. * $p < 0.05$; ** $p < 0.01$. Error bars = standard error of the mean.

Supplementary Figure 2. Analysis of apoptosis in ReNcell VM cells treated with 1μM doxycycline using Annexin V-Biotin (Streptavidin APC) and Propidium Iodide. Dot Plot of untreated 'No Doxycycline' and treated '1μM Doxycycline' ReNcell VM after 12 hours. Shown are Viable cells in lower left quadrant, Early Apoptotic in lower right quadrant (Annexin V Biotin/Streptavidin APC 647-A positive), Late Apoptotic in upper right quadrant (Annexin V Biotin/Streptavidin APC 647-A positive and Propidium Iodide positive). Cells were treated with 1μM Doxycycline for 12hrs before collecting for analysis of Early Apoptotic events. Values represent percentage of cells in each quadrant. Change in percentage of early apoptotic cells after treatment are as follows: Wildtype (0.48%), Scrambled (0.93%), 2193 (1.56%) and 4774 (3.18%).

Supplementary Figure 3. USP9X depletion after 72hrs does not result in increased apoptosis or differentiation. **(A)** Immunoblot analysis of Caspase-3 levels in USP9X depleted ReNcell VM cells after 72hrs 1μM Doxycycline treatment. **(B)** Immunoblot for βIII-tubulin and GFAP in USP9X depleted ReNcell VM after 72hrs 1μM doxycycline.

Supplementary Figure 4. Immunoblot analysis of whole cell lysate showing expression levels of G1-S Phase cell cycle proteins in USP9X depleted ReNcell VM cells 24hrs, 36hrs and 72hrs after 1μM Doxycycline addition. No change in Cyclin D1, E2F1 or total Retinoblastoma protein (Rb) protein expression in USP9X depleted lines was observed. Reduction of phosphorylated Rb (S780) in USP9X depleted

ReNcell VM cells was observed after 72hrs. All loading controls are 55kDa β-Tubulin

Supplementary Figure 5. mTOR and Rictor levels are unaltered in the absence of USP9X. **(A-C)** Densitometric analysis of p-mTOR and t-mTOR protein levels presented in Figure 3C. **(D)** Immunoblot analysis of Rictor protein in wildtype, scrambled, 2193 and 4474 cells in the presence or absence of doxycycline. β-tubulin indicates similar amounts of protein present in each lane.

Supplementary Figure 6. Reduction of RAPTOR expression in USP9x depleted ReNcells after EGF/FGF stimulation of 0, 5 and 15mins. **(A)** and **(B)** Two biological replicates of EGF/FGF stimulation experiment demonstrating reduction in RAPTOR protein levels in USP9X depleted ReNcell VM cells. **(C)** Representative reduction of USP9X depletion after 72hrs doxycycline treatment. Panel (A) and Panel (C) are from the same biological replicate.

Supplementary Figure 7. Ectopic expression of USP9X in HEK293 cells increases RAPTOR without affecting pS6 levels. Densitometric analysis of Raptor **(A),** pS6 and t-S6 levels **(B-D)** in proliferating HEK293 cells following USP9X transfection as presented in Figure 4C. **(E)** Densitometric analysis of RAPTOR levels following WP1130 presented in Figure 3D.

Supplementary Figure 8. Densitometric analysis of RAPTOR protein levels in USP9X shRNA treated ReNcell VM cells in the presence of proteasome inhibitor epoxomicin. Raptor level increases in presence of epoxomicin. Depletion of USP9X (+DOX) decreases RAPTOR levels in both presence and absence of epoxomicin. Analysis of three immunoblots (2193 and 4774 in Figure 4D, plus additional 4774 replicate). Statistical significance assessed by Student's t-test. *, $p < 0.05$.

Supplementary Figure 9. USP9X depletion does not affect Raptor gene expression. Quantitative RT-PCR analysis of ReNcell VM cells treated with doxycycline (+DOX) did not detect any change in Raptor mRNA level, relative to GAPDH, in control (WT, Scr) or USP9X depleted (2194, 4774) cells. Bars represent SEM.

Supplementary Figure 10. Neural progenitor proliferation is decreased in neurospheres derived from Usp9x$^{-/Y}$ brains. Neurospheres were exposed to a 6h EdU pulse before Flow cytometry analysis. Fewer EdU labelled cells were detected in neurospheres from Usp9x$^{-/Y}$ mice. *, $p<0.05$.

Supplementary Figure 11 Full length immunoblots corresponding to blots presented in Figure 3.

Supplementary Figure 12. Full length immunoblots corresponding to blots presented in Figure 4.

Supplementary Figure 13. Full length immunoblots corresponding to blots presented in Figure 4D.

Supplementary Figure 14. Full length immunoblots corresponding to blots presented in Figure 6.

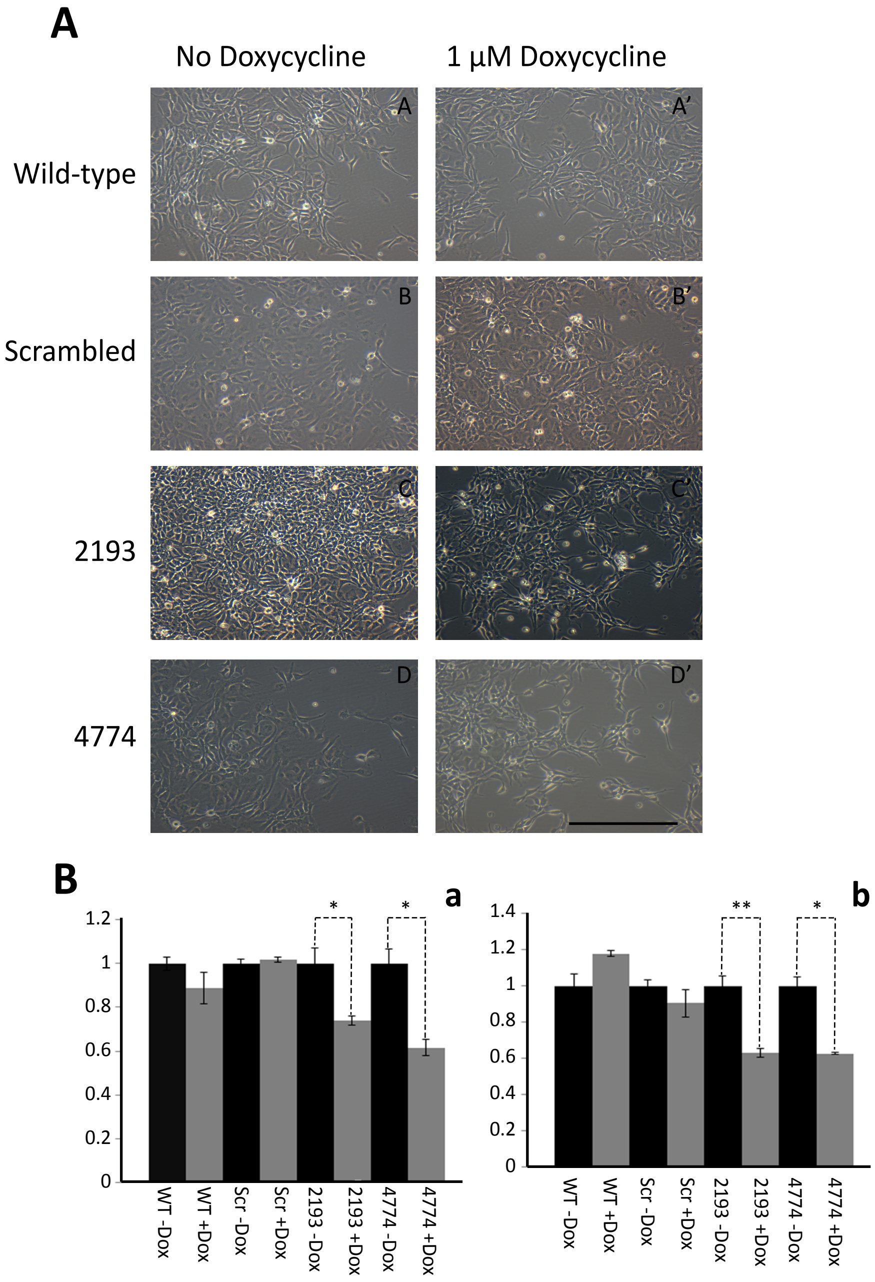

Wood Supplementary Figure 1

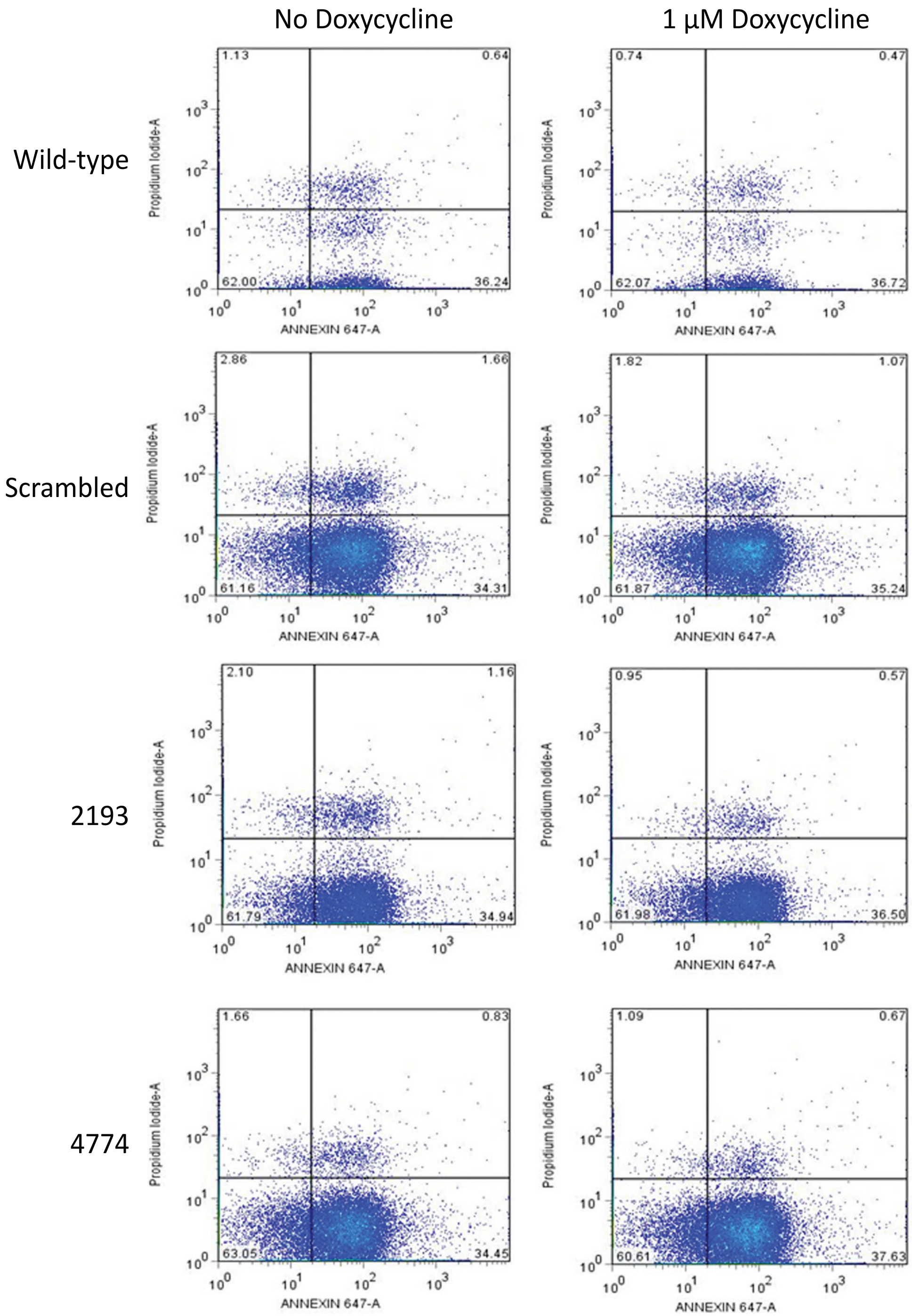

Wood Supplementary Figure 2

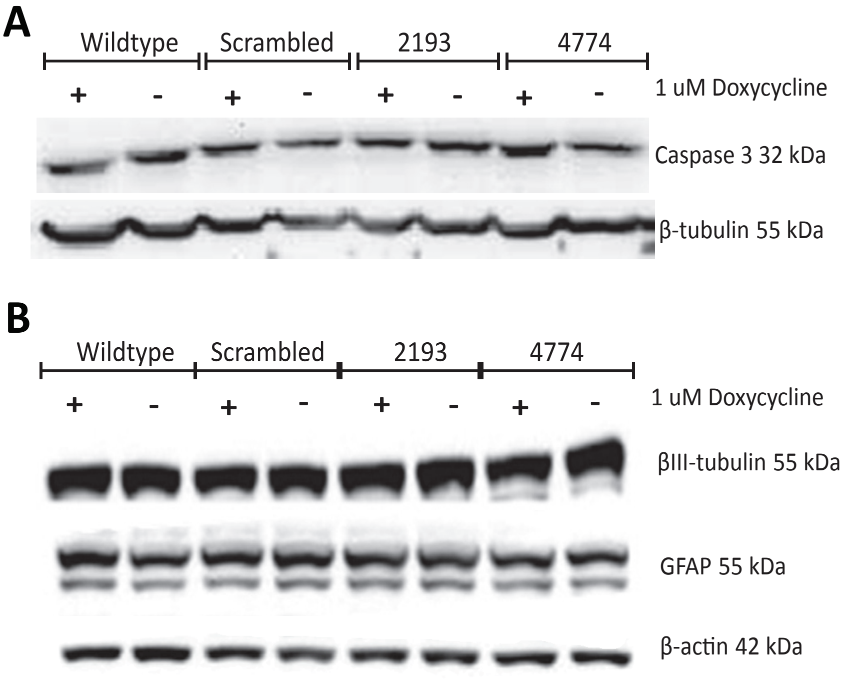

Wood Supplementary Figure 3

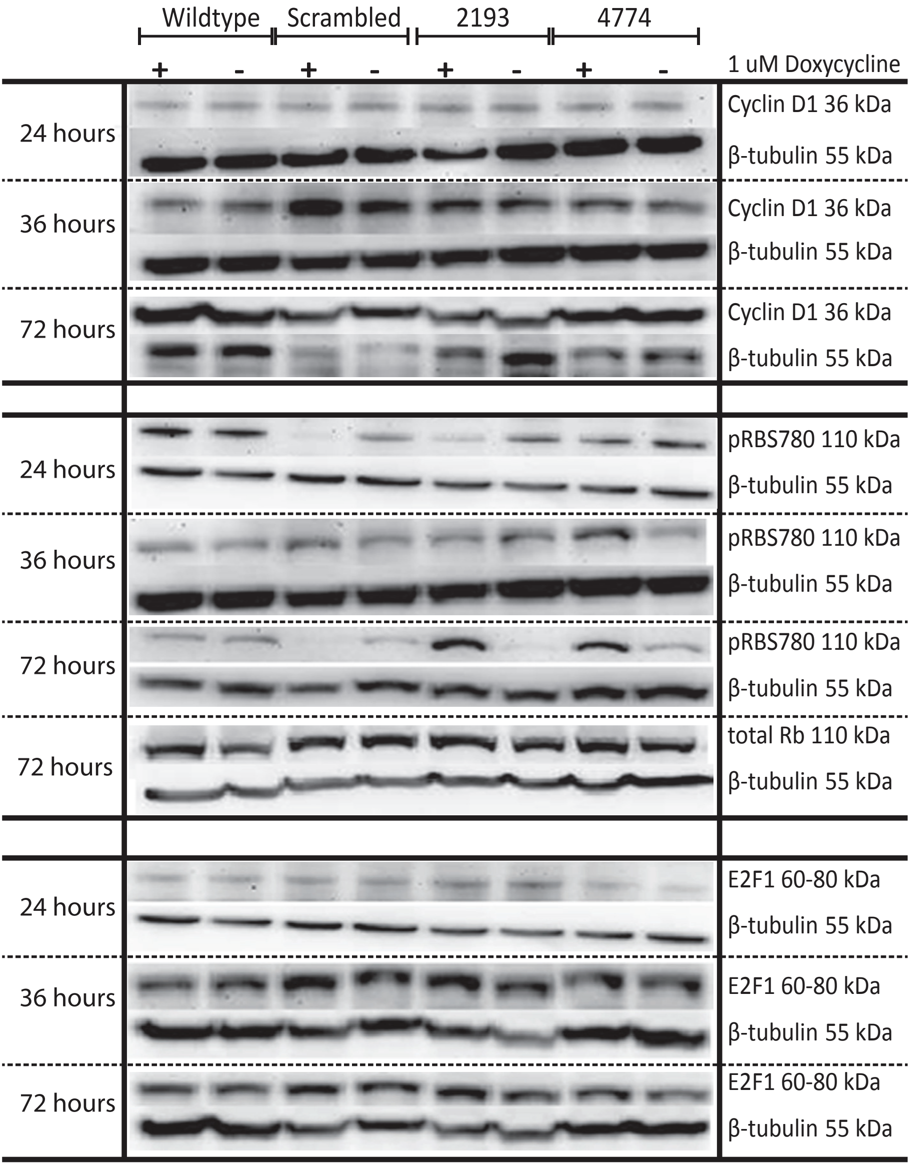

Wood Supplementary Figure 4

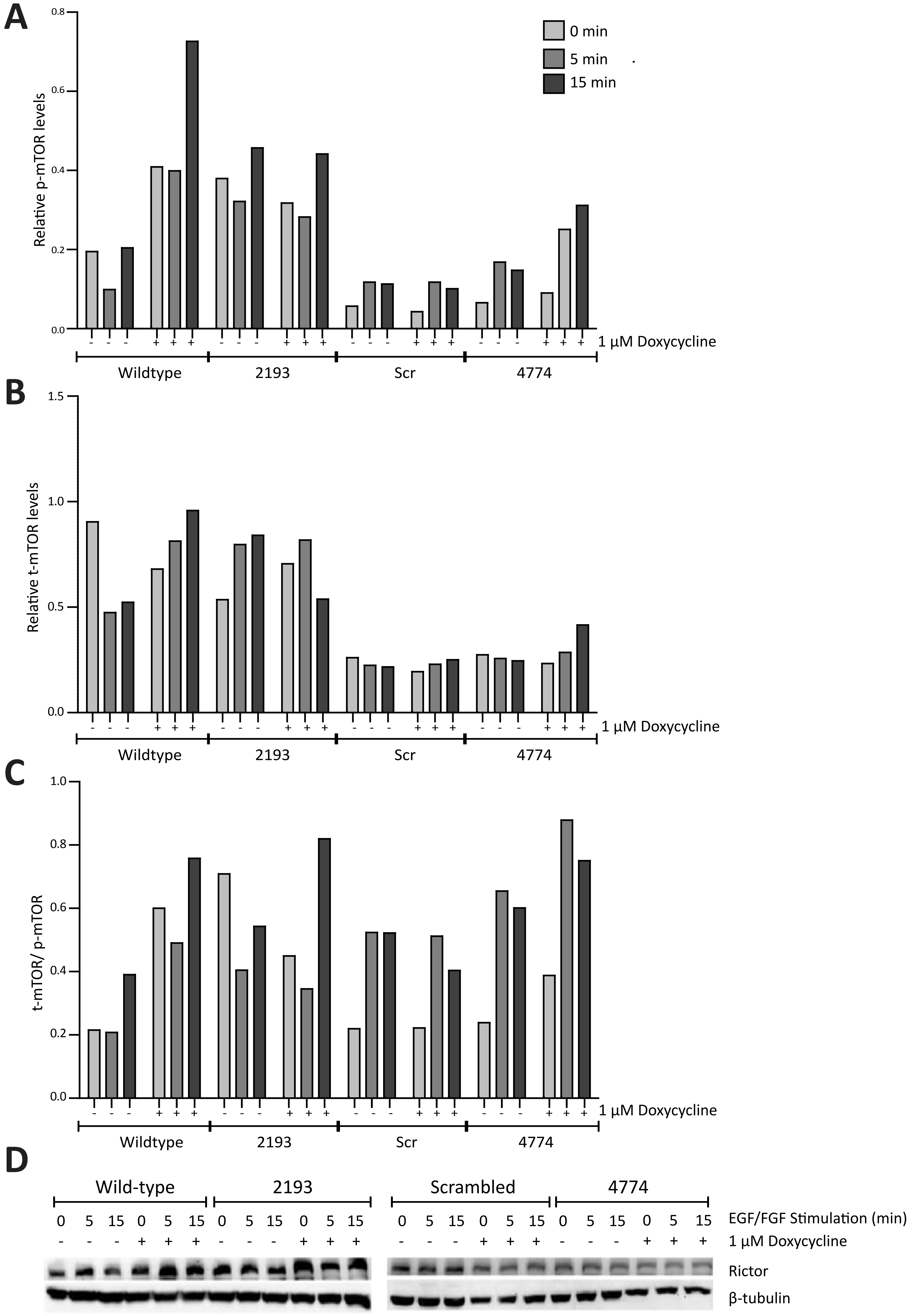

Wood Supplementary Figure 5

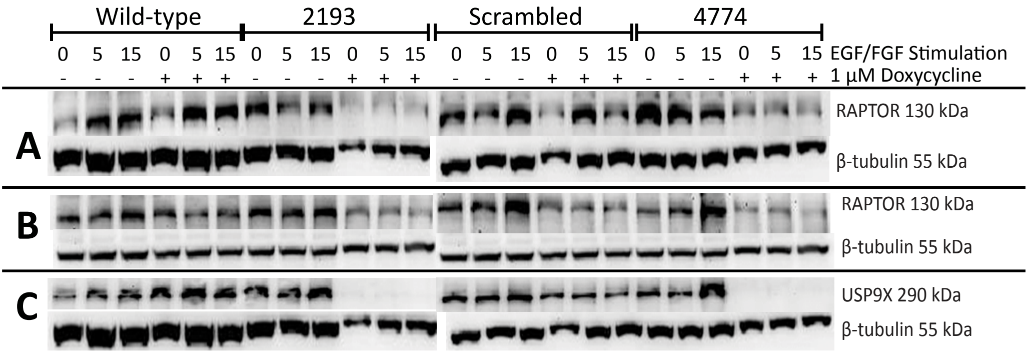

Wood Supplementary Figure 6

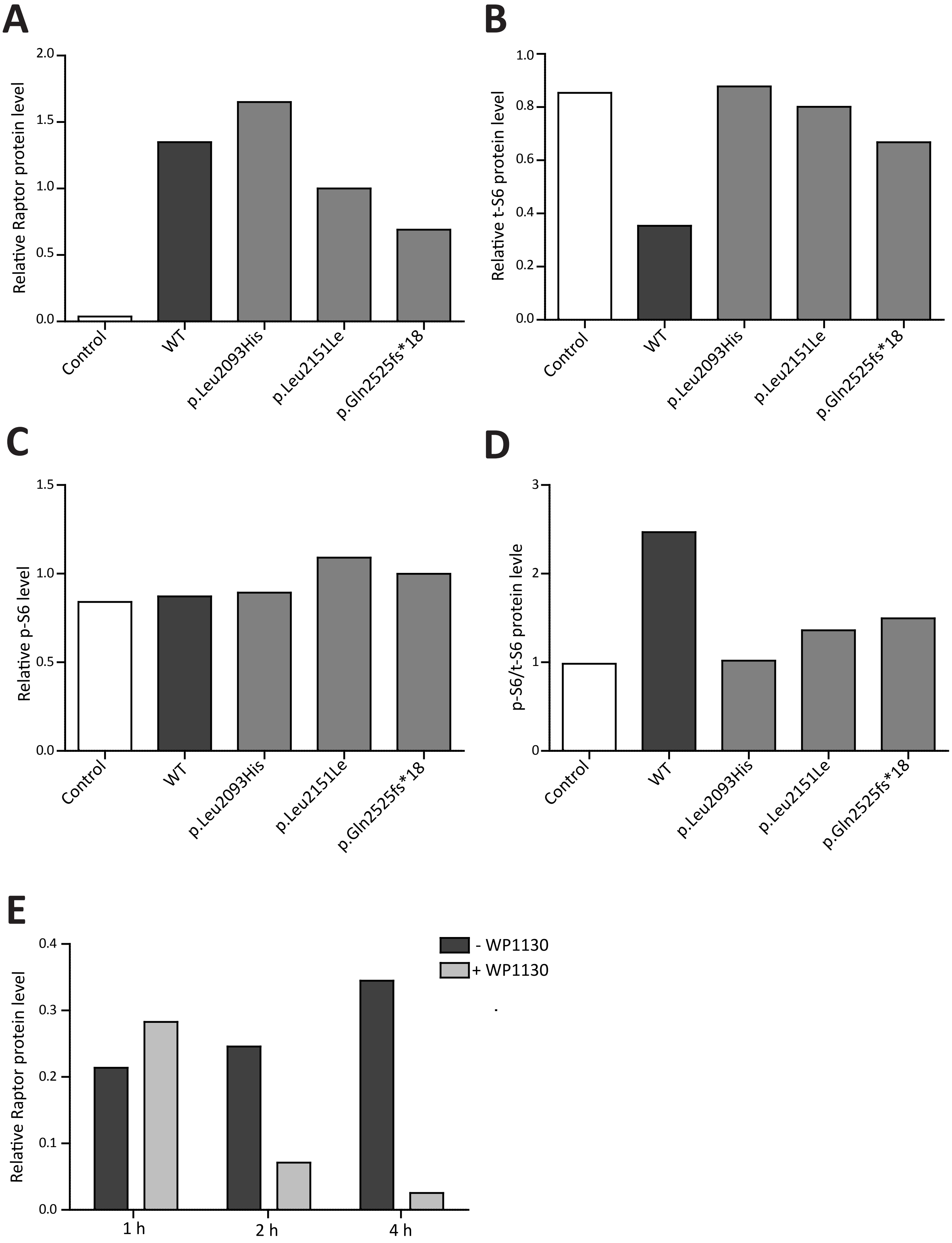

Wood Supplementary Figure 7

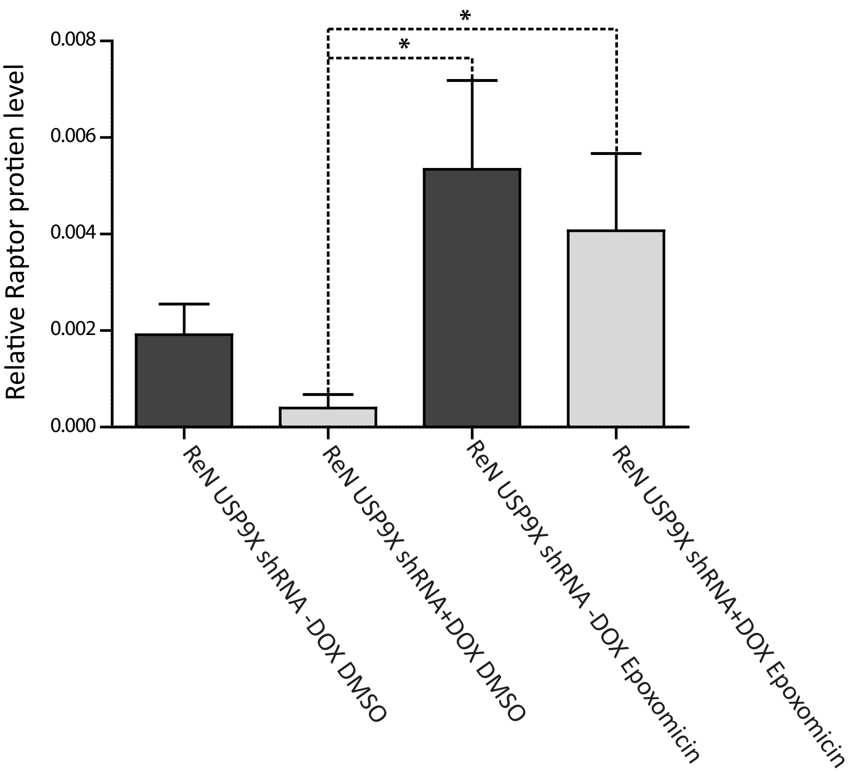

Wood Supplementary Figure 8

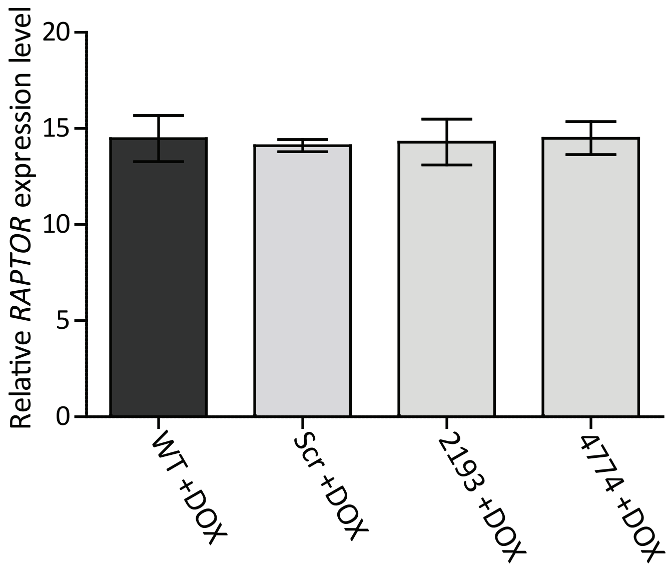

Wood Supplementary Figure 9

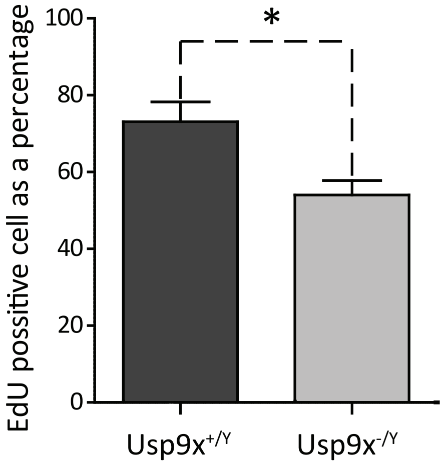

Wood Supplementary Figure 10

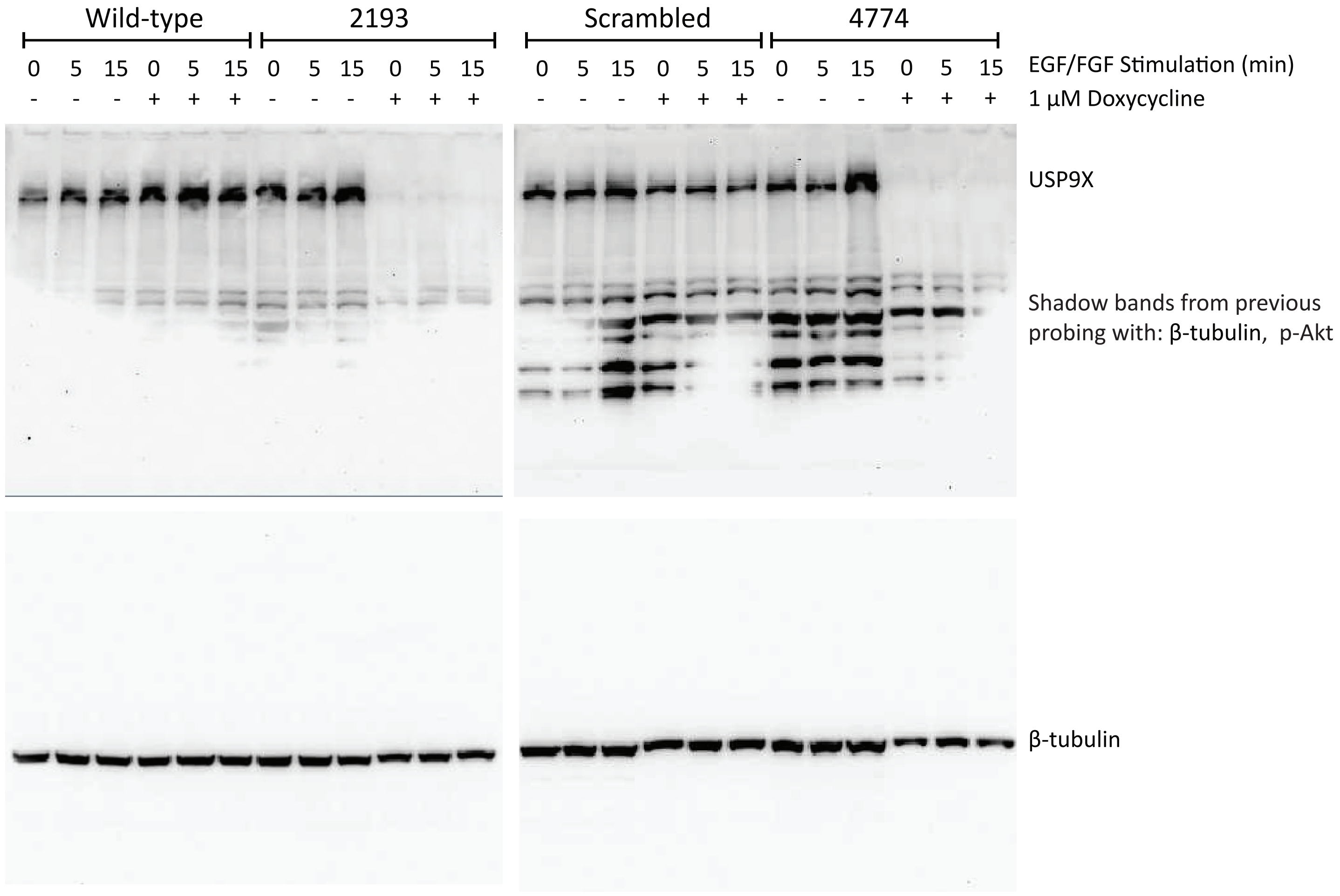

Wood Supplementary Figure 11A - to match Figure 3A full length USP9X and β-tubulin blots (bottom)

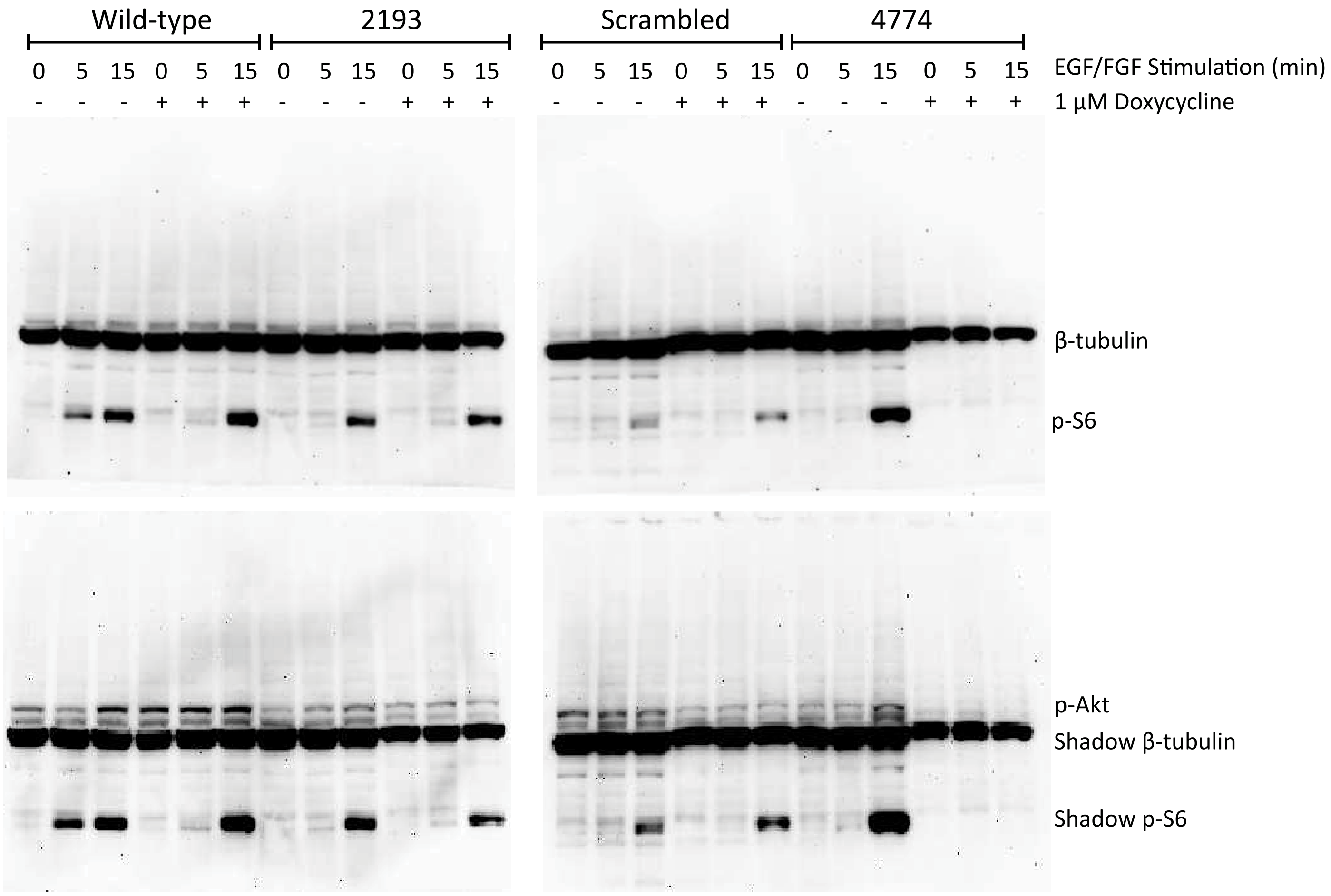

Wood Supplementary Figure 11B - to match Figure 3A full length p-S6, p-Akt and β-tubulin blots

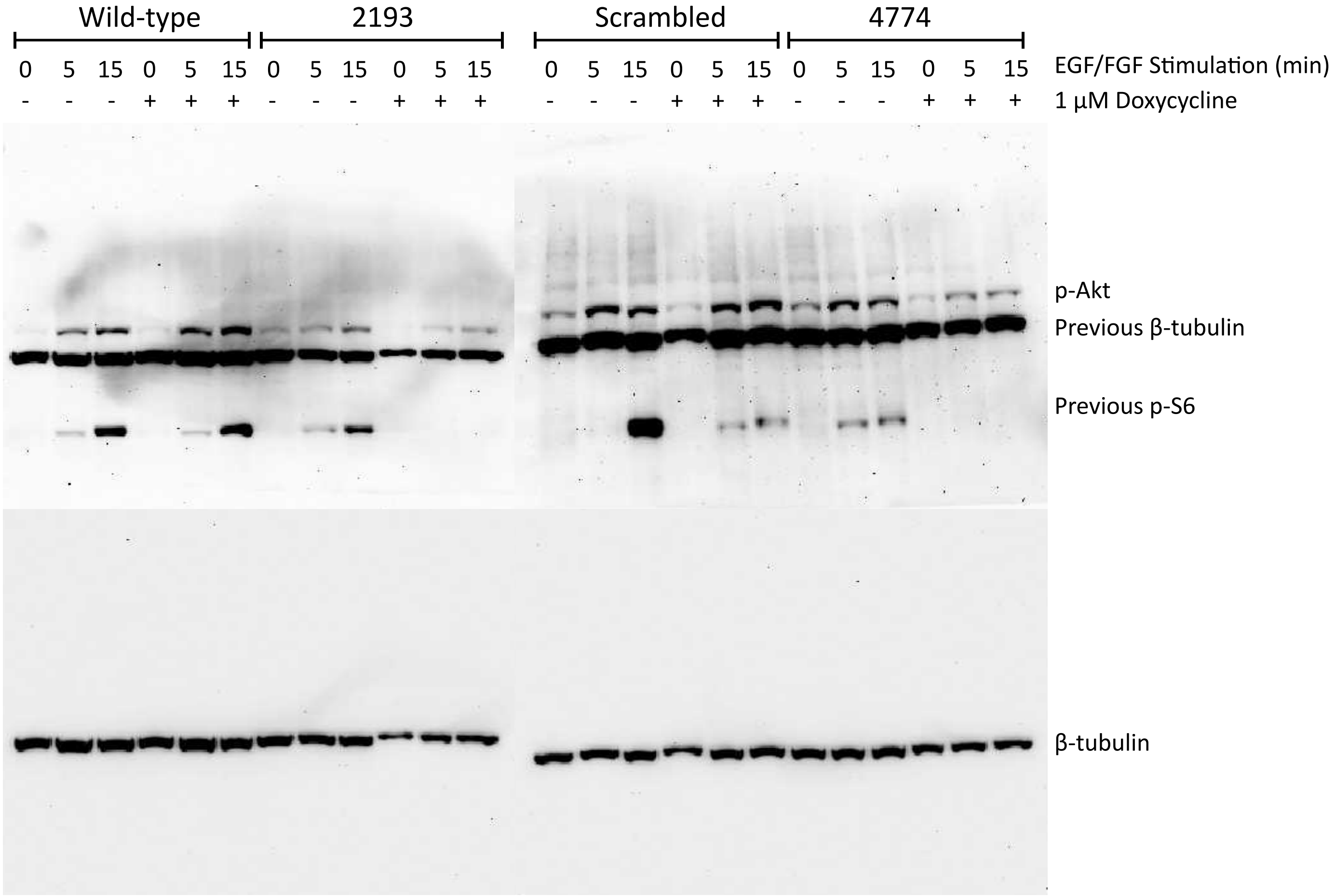

Wood Supplementary Figure 11C - to match Figure 3A full length p-Akt and β-tubulin blots

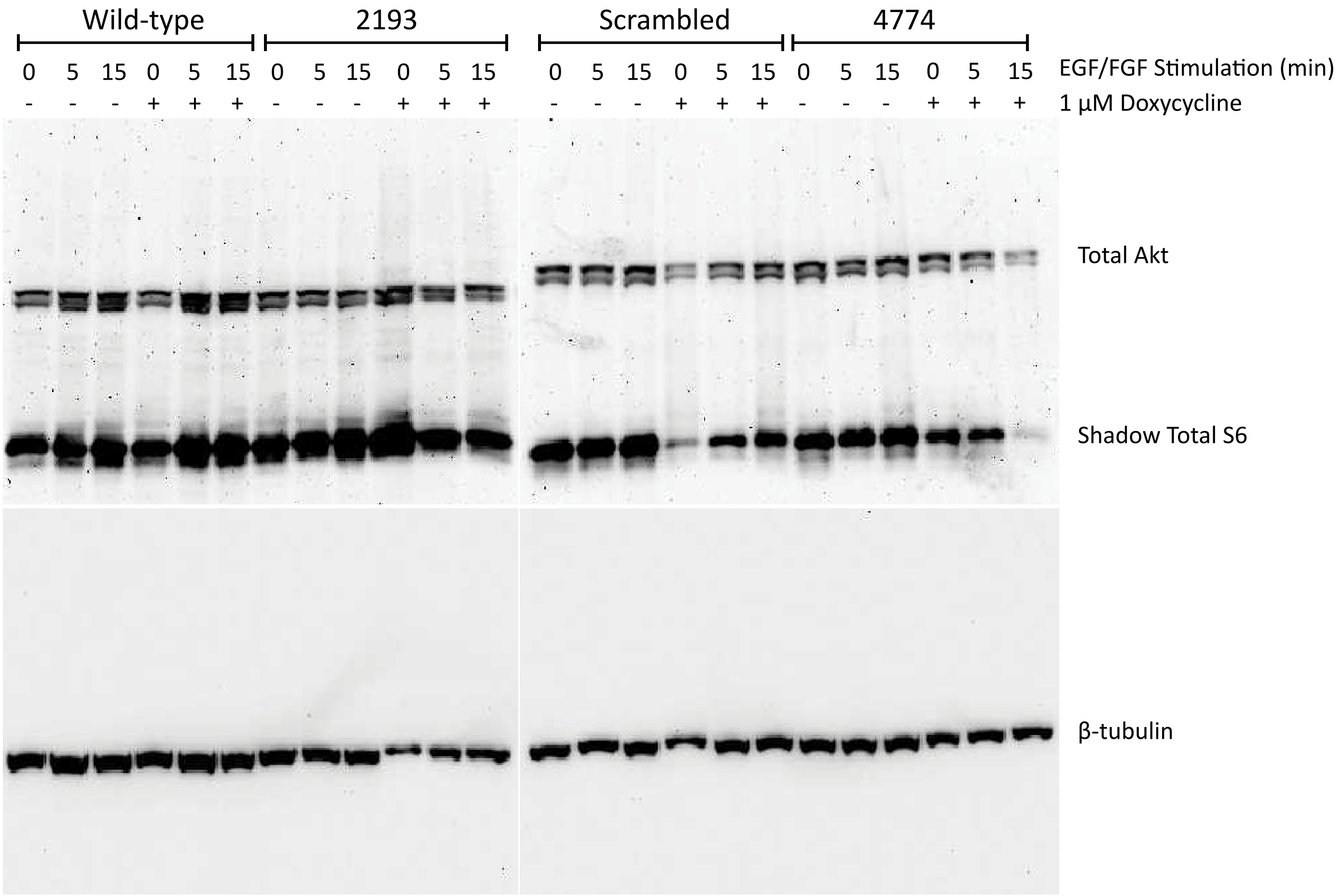

Wood Supplementary Figure 11D - to match Figure 3A full length Total Akt, Total S6 and β-tubulin blots

24 hrs Time point

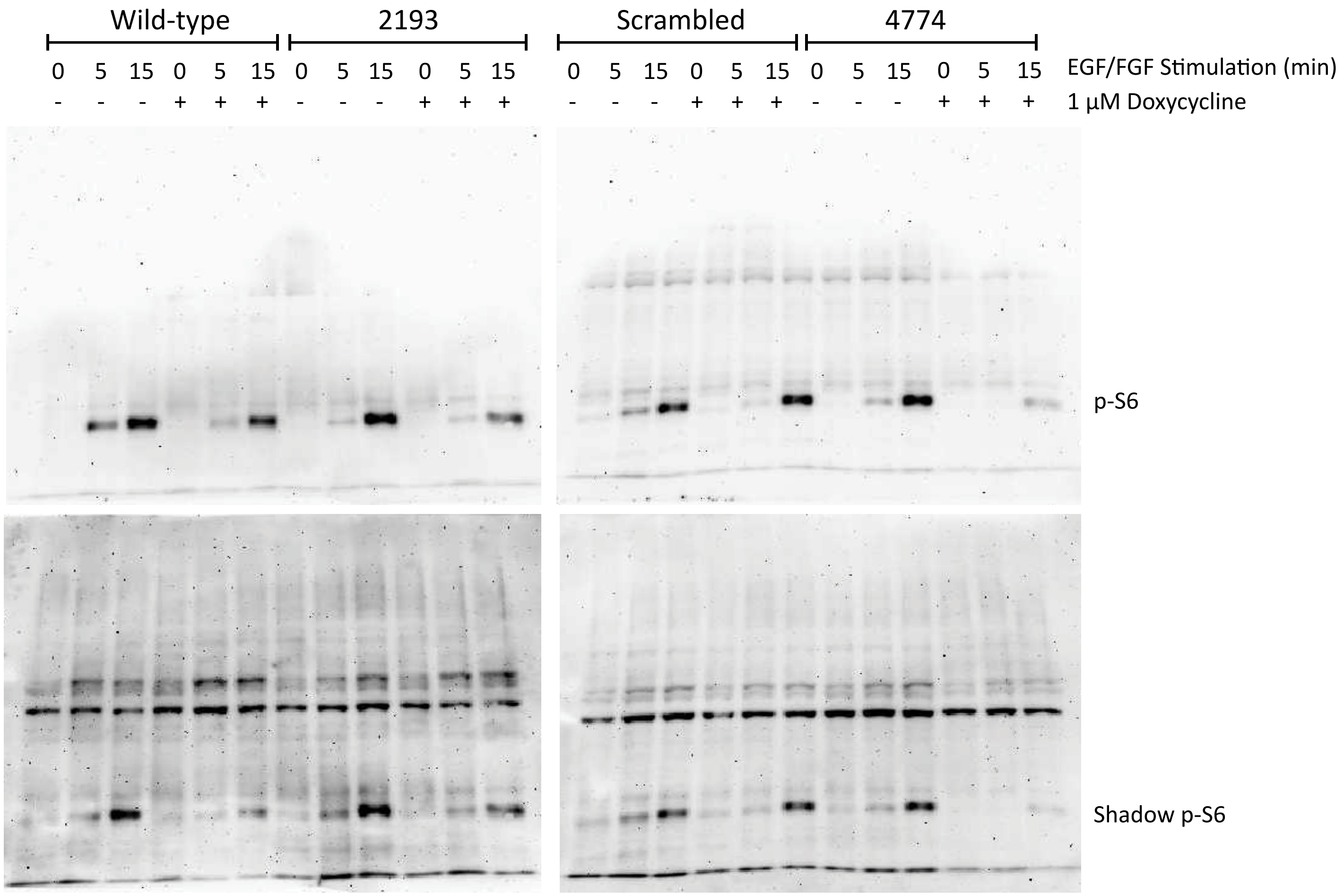

Wood Supplementary Figure 11E - to match Figure 3B full length p-S6 and β-tubulin blots

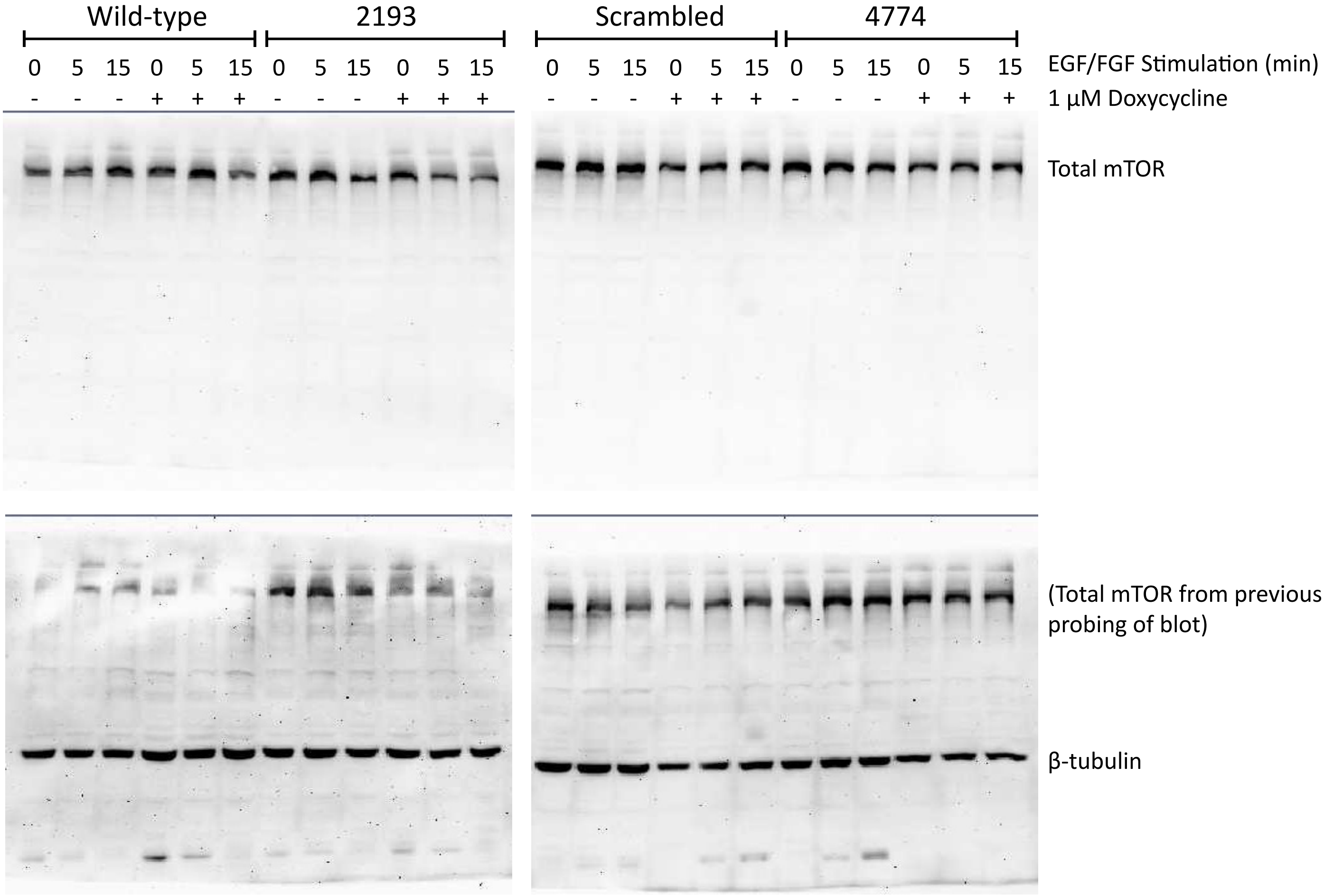

Wood Supplementary Figure 11F - to match Figure 3C full length Total mTOR and β-tubulin blots

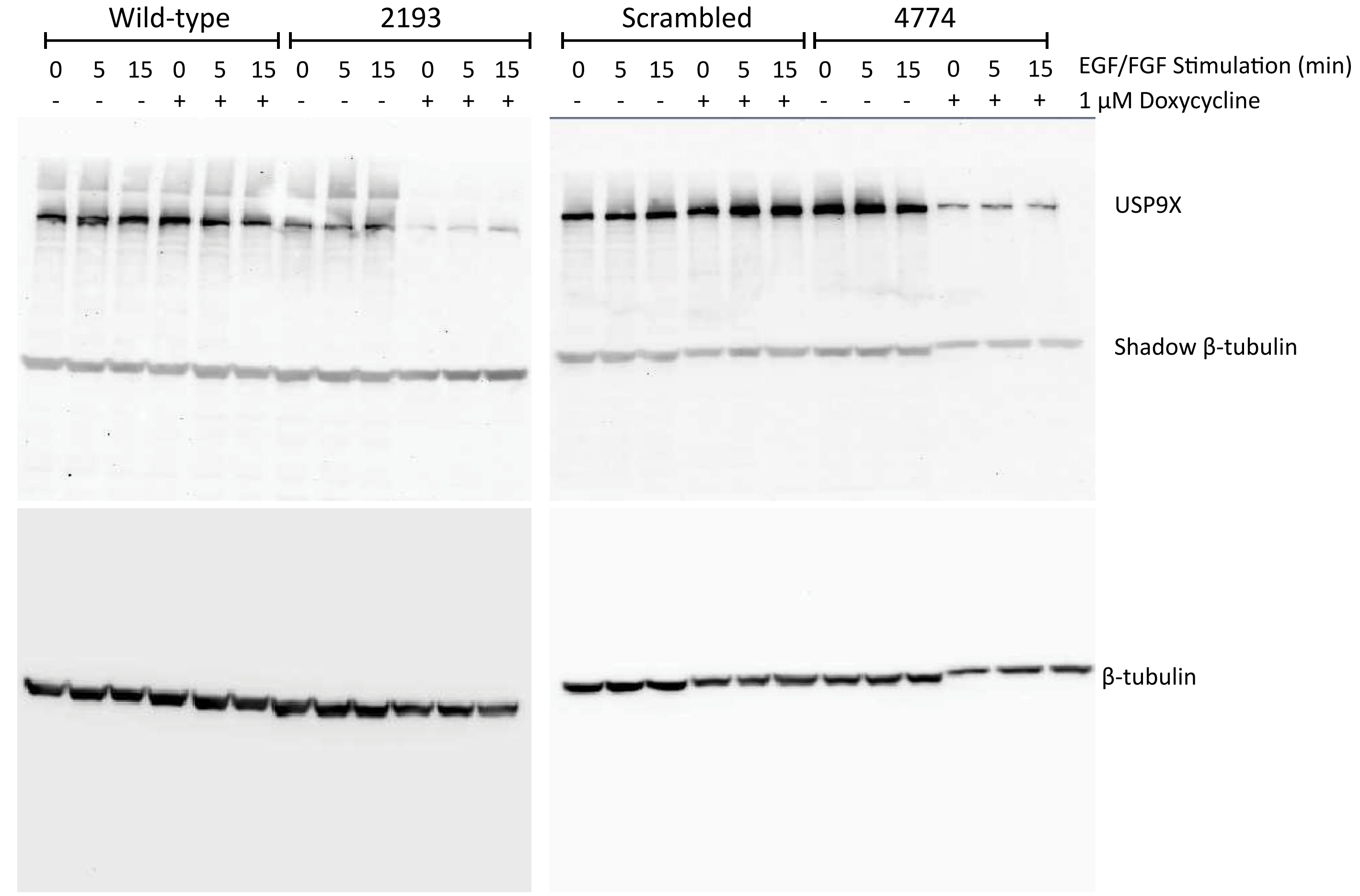

Wood Supplementary Figure 11G - to match Figure 3C full length USP9X and β-tubulin blots (bottom)

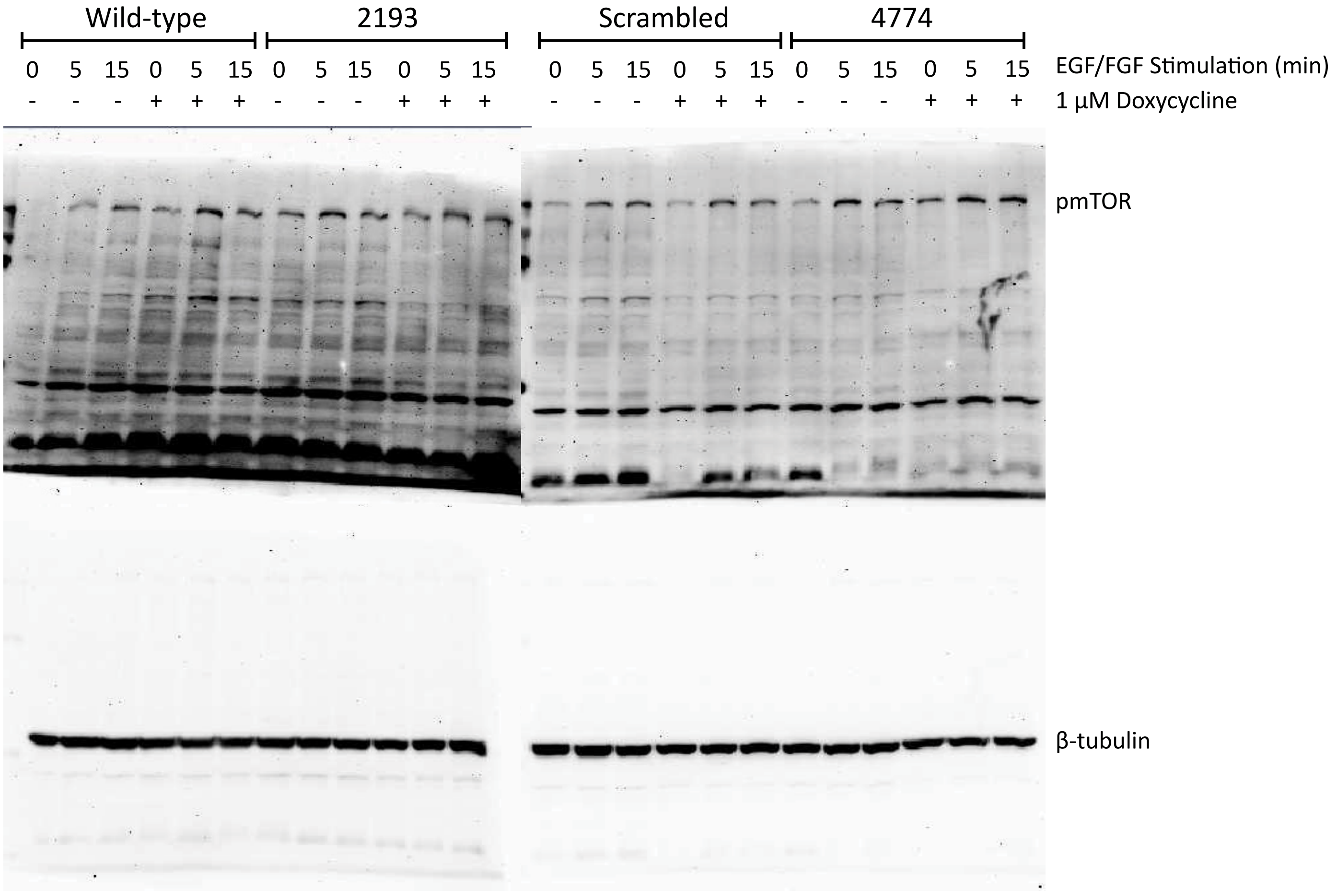

Wood Supplementary Figure 11H - to match Figure 3C full length pmTOR and β-tubulin blots (bottom)

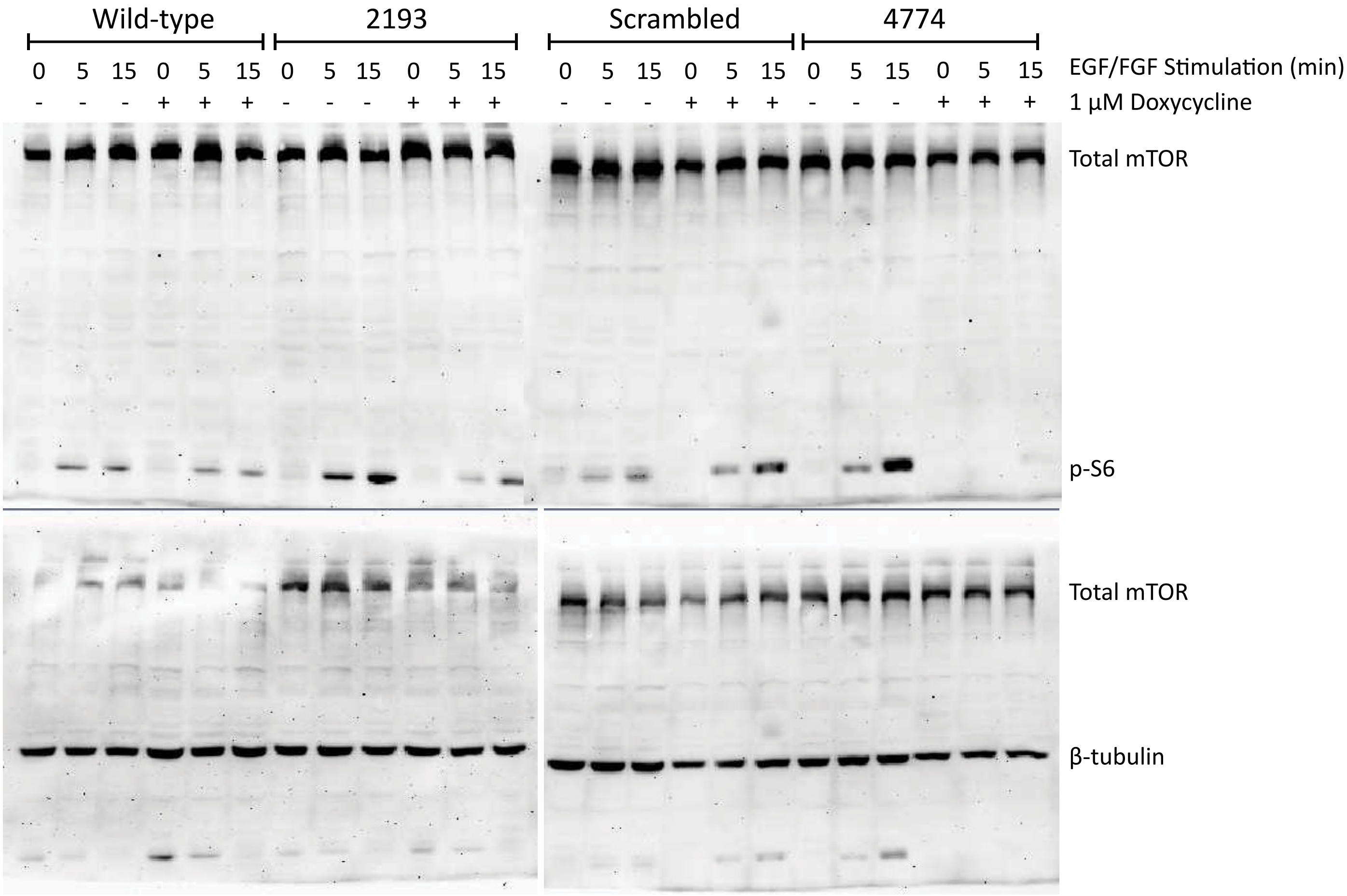

Wood Supplementary Figure 11I - to match Figure 3C full length Total mTOR and β-tubulin blots (bottom)

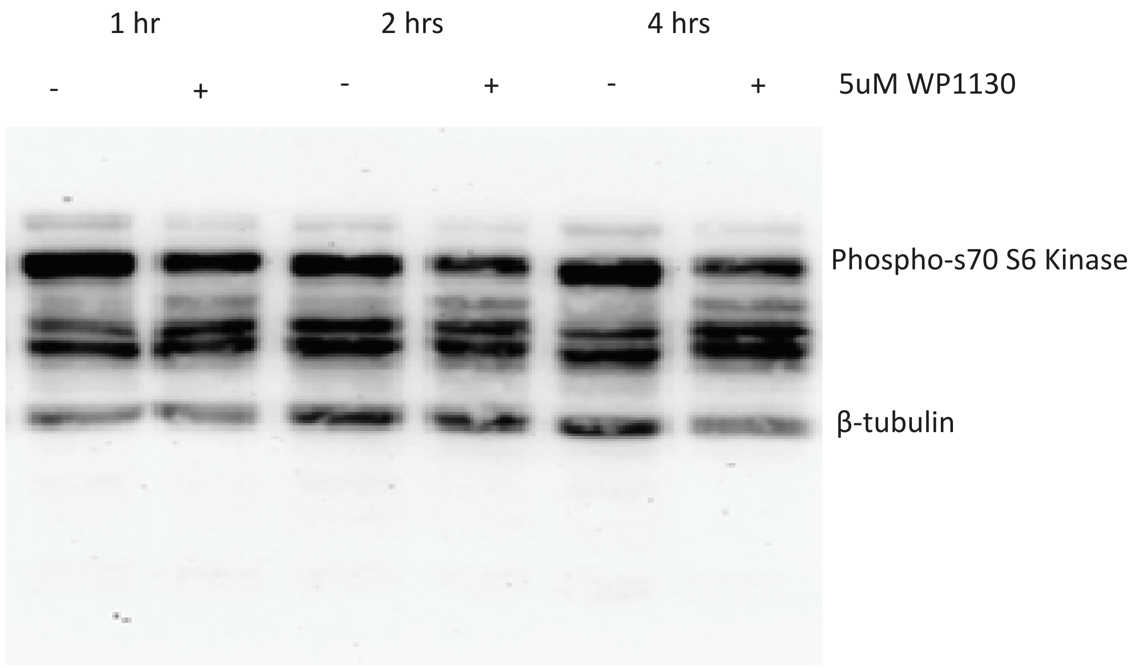

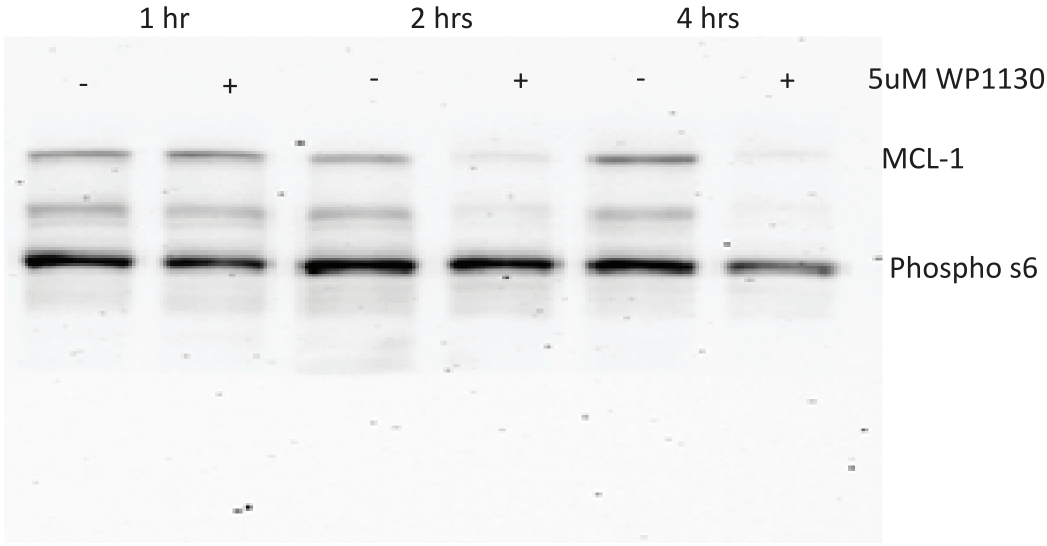

Wood Supplementary Figure 12A - to match Figure 4B full length blots of Phospho-s70, MCL-1 and β-tubulin

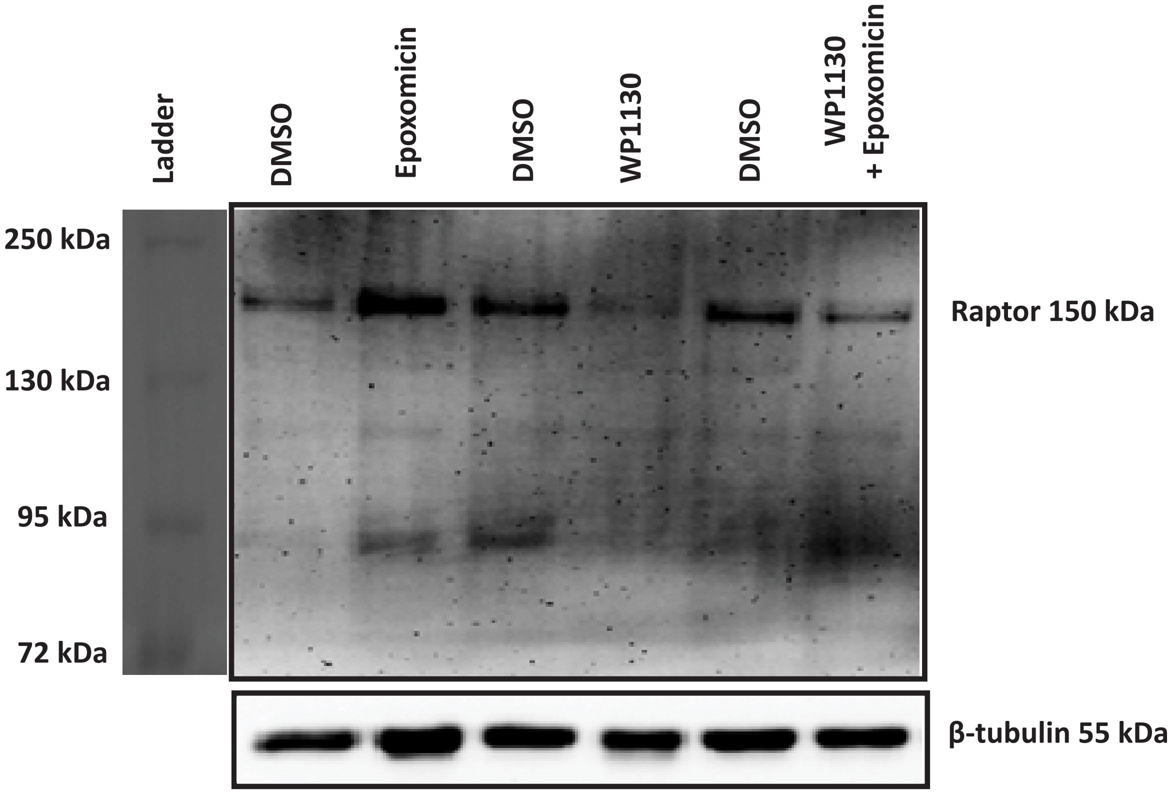

Wood Supplementary Figure 12B - to match FIgure 4C full length blot for Raptor

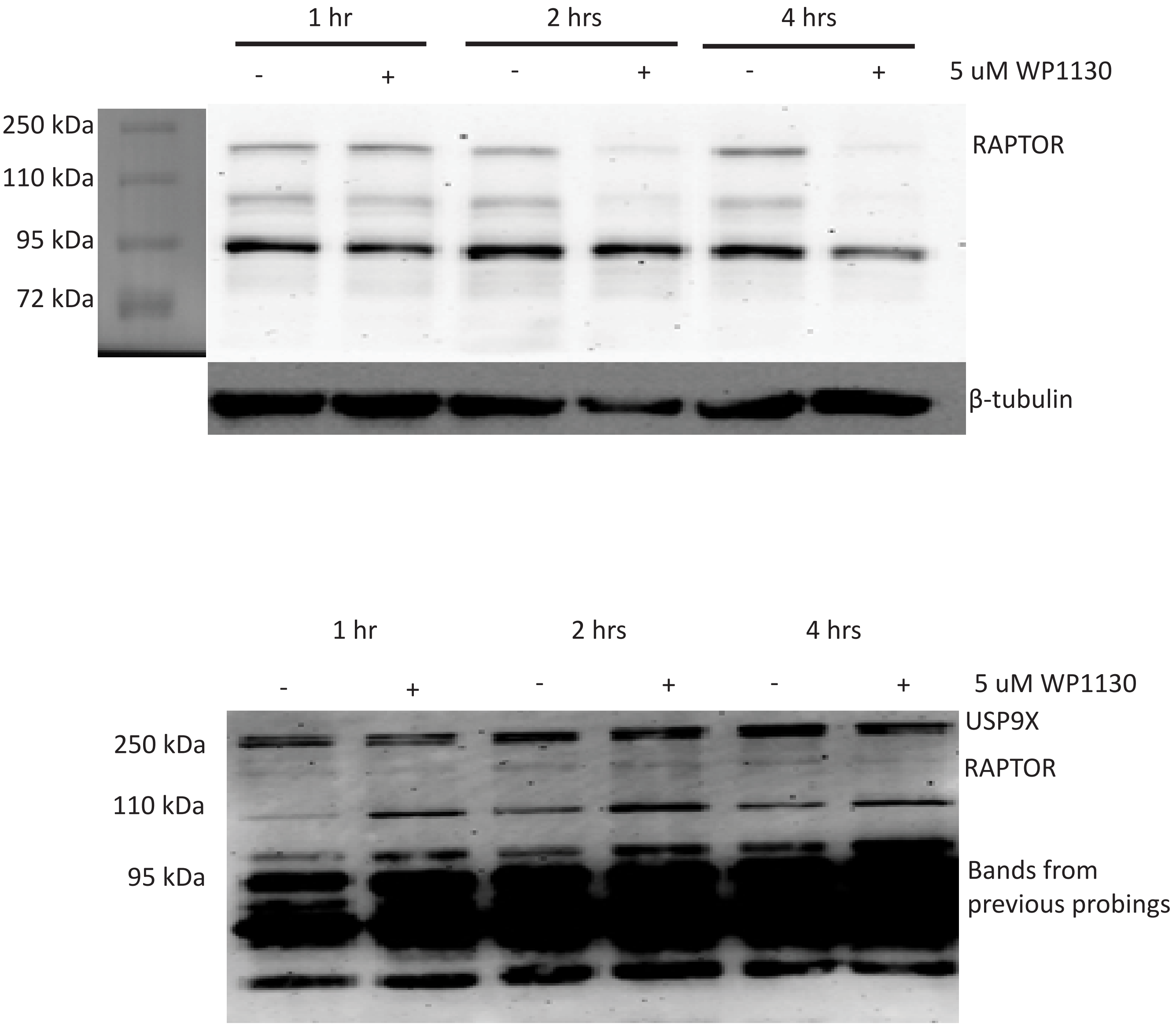

Wood Supplementary Figure 12C - to match FIgure 4D full length blot of Raptor, USP9X and β-tubulin

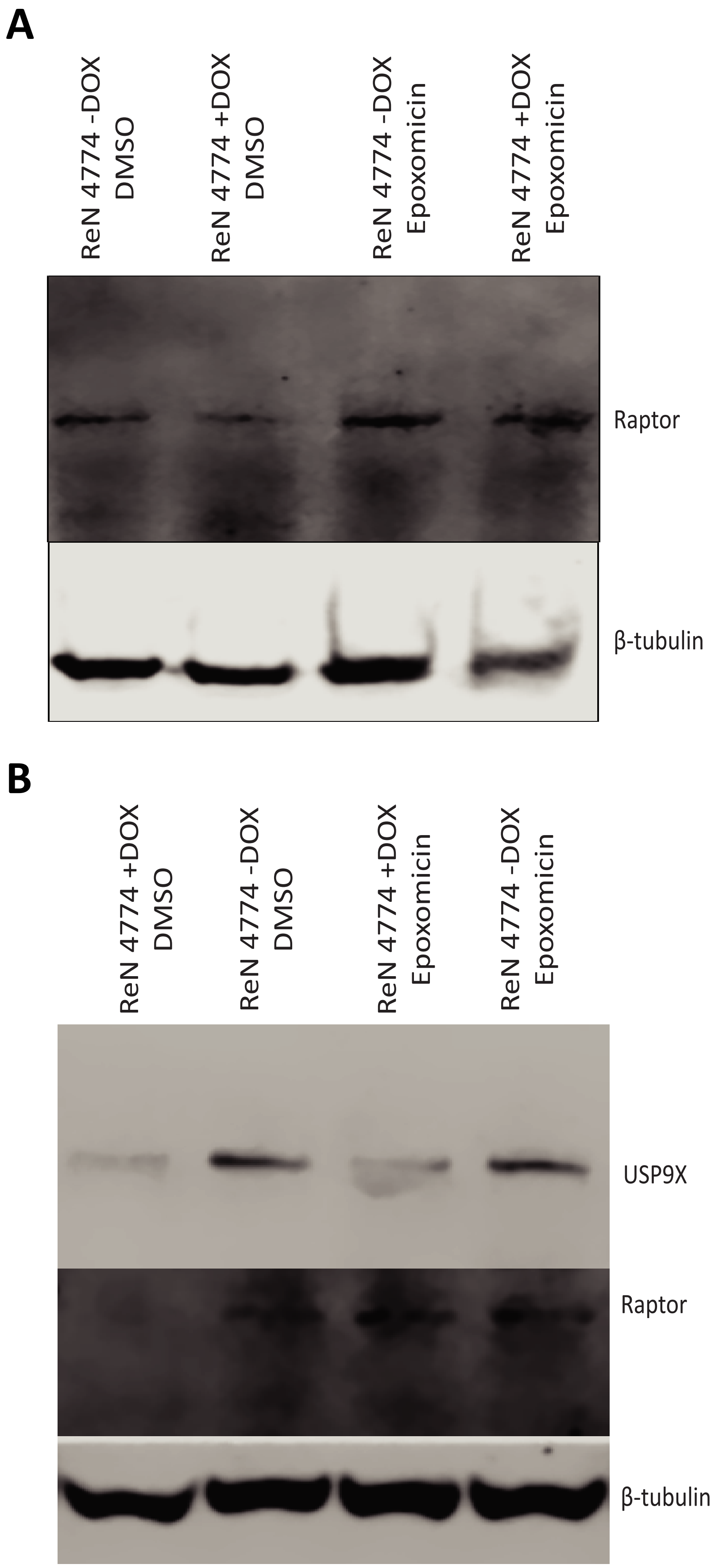

Wood Supplementary Figure 13A, B - to match FIgure 4D full length blot of Raptor, USP9X and β-tubulin (A) and the second biological replicate (B).

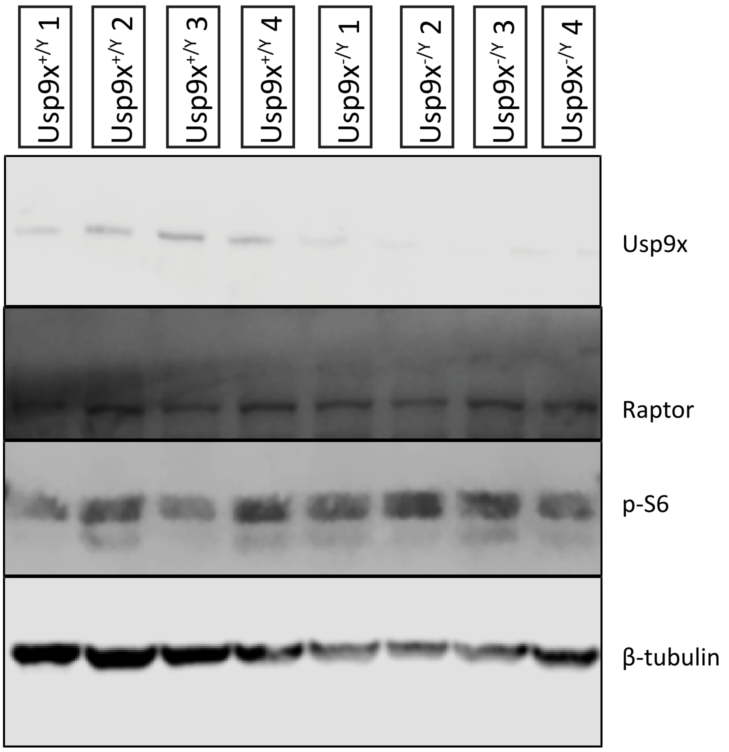

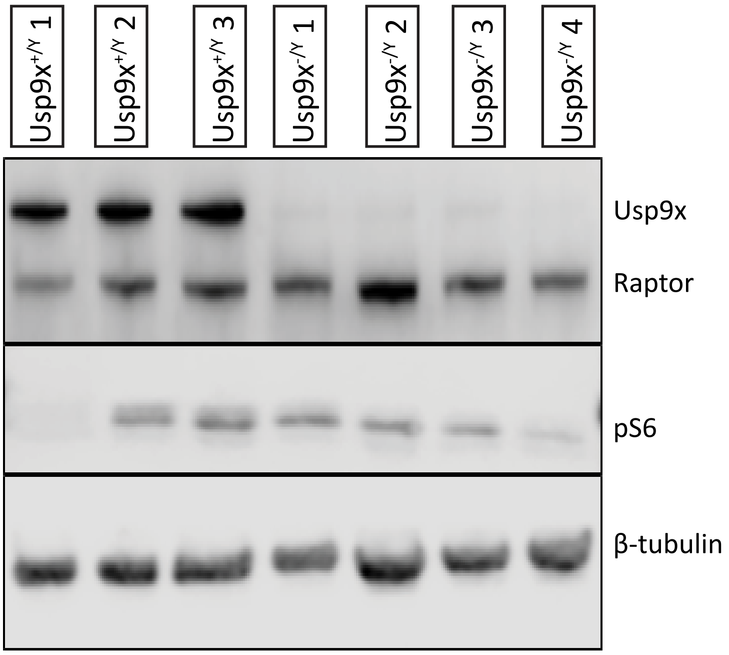

Wood Supplementary Figure 14
